# Green Synthesis, Characterization, and Potential Antibacterial and Anticancer Applications of Gold Nanoparticles: Current Status and Future Prospects

**DOI:** 10.3390/biomedicines13051184

**Published:** 2025-05-13

**Authors:** Md. Amdadul Huq, Md. Rasel Rana, Abdus Samad, Md. Shahedur Rahman, M. Mizanur Rahman, Md Ashrafudoulla, Shahina Akter, Jong-Whi Park

**Affiliations:** 1 Department of Life Sciences, College of BioNano Technology, Gachon University, Seongnam 13120, Republic of Korea; 2 Department of Microbiology, Faculty of Science and Engineering, Rabindra Maitree University, Kushtia 7000, Bangladesh; raselrana8659@gmail.com; 3 Graduate School of Biotechnology, College of Life Science, Kyung Hee University, Yongin 17104, Republic of Korea; kazisamad50@gmail.com; 4 Department of Genetic Engineering and Biotechnology, Jashore University of Science and Technology, Jashore 7408, Bangladesh; ms.rahman@just.edu.bd; 5 Department of Biotechnology and Genetic Engineering, Faculty of Biological Science, Islamic University, Kushtia 7003, Bangladesh; mmrahmanbtg79@hotmail.com; 6 Department of Food Science, Center for Food Safety, University of Arkansas System Division of Agriculture, Fayetteville, AR 72704, USA; mda@uark.edu; 7 Department of Food Science and Biotechnology, Gachon University, Seongnam 13120, Republic of Korea; shahinabristy16@gmail.com

**Keywords:** gold nanoparticles, green synthesis, characterization, antibacterial applications, anticancer applications

## Abstract

Drug resistance is a serious problem for human health worldwide. Day by day this drug resistance is increasing and creating an anxious situation for the treatment of both cancer and infectious diseases caused by pathogenic microorganisms. Researchers are trying to solve this terrible situation to overcome drug resistance. Biosynthesized gold nanoparticles (AuNPs) could be a promising agent for controlling drug-resistant pathogenic microorganisms and cancer cells. AuNPs can be synthesized via chemical and physical approaches, carrying many threats to the ecosystem. Green synthesis of AuNPs using biological agents such as plants and microbes is the most fascinating and attractive alternative to physicochemical synthesis as it offers many advantages, such as simplicity, non-toxicity, cost-effectiveness, and eco-friendliness. Plant extracts contain numerous biomolecules, and microorganisms produce various metabolites that act as reducing, capping, and stabilizing agents during the synthesis of AuNPs. The characterization of green-synthesized AuNPs has been conducted using multiple instruments including UV–Vis spectrophotometry (UV–Vis), transmission electron microscopy (TEM), scanning electron microscopy (SEM), atomic force microscopy (AFM), X-ray diffraction (XRD), DLS, and Fourier transform infrared spectroscopy (FT-IR). AuNPs have detrimental effects on bacterial and cancer cells via the disruption of cell membranes, fragmentation of DNA, production of reactive oxygen species, and impairment of metabolism. The biocompatibility and biosafety of synthesized AuNPs must be investigated using a proper in vitro and in vivo screening model system. In this review, we have emphasized the green, facile, and eco-friendly synthesis of AuNPs using plants and microorganisms and their potential antimicrobial and anticancer applications and highlighted their antibacterial and anticancer mechanisms. This study demonstrates that green-synthesized AuNPs may potentially be used to control pathogenic bacteria as well as cancer cells.

## 1. Introduction

Drug resistance has become a global challenge for human health, leading to increased morbidity, mortality, and economic burden. In the realm of cutting-edge scientific advancements, the synthesis and utilization of nanomaterials have emerged as a revolutionary avenue with profound implications for medical and healthcare applications [1]. Nanoparticles are small particles and have significant applications in the healthcare and environmental sectors. They play an important role in drug development, drug delivery, tissue engineering, wound healing, water treatment, soil remediation, air pollution control, etc. [2,3,4]. Nanoparticles can be synthesized via chemical and physical approaches, carrying many threats to the ecosystem. The main drawbacks of physicochemical methods of nanoparticle synthesis are the potential use of hazardous chemicals, generation of hazardous byproducts, high energy consumption, and high cost. The green synthesis of nanoparticles using biological agents such as plants and microbes is the most fascinating and attractive alternative to physicochemical synthesis as it offers many advantages such as simplicity, non-toxicity, cost-effectiveness, and eco-friendliness. Plant extracts contain numerous biomolecules, such as alkaloids, flavonoids, terpenoids, phenolic compounds, etc., that act as reducing, capping, and stabilizing agents during the synthesis of nanoparticles. Similarly, microorganisms produce various primary and secondary metabolites, such as amino acids, enzymes, flavonoids, etc., that serve as reducing, capping, and stabilizing agents for the synthesis of nanoparticles. These plants and microbe-derived biomolecules facilitate the reduction of gold ions into gold nanoparticles (AuNPs), prevent aggregation, and stabilize the final nanoparticles. Notably, the green synthesis of nanoparticles has garnered significant attention due to its eco-friendly and sustainable approach. Nanoparticles synthesized using natural compounds derived from plants, bacteria, or fungi, hold immense promise in various fields, notably in the domains of anticancer and antimicrobial treatments [4,5,6,7,8].

The convergence of nanotechnology and green synthesis has paved the way for a new era of biomedical research and development [9,10]. In the fight against cancer, conventional treatments often come with severe side effects and limitations. However, the advent of green-synthesized nanoparticles offers a potential solution. These nanoparticles, synthesized using plant extracts rich in bioactive compounds, exhibit remarkable anticancer properties [11,12]. By targeting cancer cells with enhanced precision and minimizing damage to healthy cells, these nanoparticles hold the potential to revolutionize cancer therapy, ushering in a new era of more effective and less toxic treatments [7,13]. At the heart of this technological leap lies the remarkable world of nanoparticles. These tiny structures, typically ranging from 1 to 100 nanometers, can be engineered to possess unique physical, chemical, and biological properties due to their quantum-size effects and high surface-to-volume ratios [14,15]. Such attributes have granted nanoparticles the ability to interact with biological systems in profound ways, offering a wide range of medical applications [5,16,17]. While the potential of nanoparticles is unquestionable, concerns regarding the environmental impact of their synthesis have prompted researchers to explore sustainable alternatives. Green synthesis of nanoparticles has emerged as a promising solution, capitalizing on the inherent properties of natural compounds to reduce the need for harsh chemicals and energy-intensive processes [7,18,19]. This approach aligns with the principles of green chemistry and provides a pathway to environmentally friendly nanoparticle production [20,21].

The battle against cancer is a major global challenge, driving relentless efforts to discover novel, effective, targeted therapies [21,22]. Green-synthesized nanoparticles have emerged as a potential game-changer in this arena. The natural compounds in their synthesis often contain bioactive agents with inherent anticancer properties. When harnessed to fabricate nanoparticles, these compounds can be directed to selectively target cancer cells while minimizing harm to healthy tissues, offering a more focused and less toxic alternative to conventional treatments [23]. Nanoparticles have detrimental effects on cancer cells via the disruption of cell membranes, fragmentation of DNA, production of reactive oxygen species, and impairment of metabolism. The unique physicochemical properties of nanoparticles enable them to penetrate cell membranes, deliver therapeutic payloads, and induce controlled cell death, thereby enhancing the efficacy of anticancer treatments [24]. The growing menace of antimicrobial resistance has underscored the urgency for innovative strategies to combat infectious diseases. Green-synthesized nanoparticles have emerged as a potential tool in the fight against drug-resistant microbes. Their small size and high surface area provide ample opportunities for interaction with microbial cells, disrupting their membranes, interfering with essential cellular processes, and impeding their proliferation [25,26]. This multi-pronged approach offers a distinct advantage, as it reduces the likelihood of developing resistance to nanoparticle-based antimicrobial agents [27]. By harnessing the power of nature’s compounds, these nanoparticles hold the potential to overcome the limitations of traditional antibiotics and contribute to the battle against infectious diseases [28,29].

Beyond their scientific significance, the economic implications of green-synthesized nanoparticles in anticancer and antimicrobial applications are considerable [30]. As research in this field advances, it has the potential to stimulate various industries, from pharmaceuticals to biotechnology, creating opportunities for innovation, job creation, and economic growth [31]. Developing novel therapies can reduce healthcare costs, as more targeted and effective treatments mitigate the need for lengthy hospital stays and extensive follow-up care [32]. Furthermore, adopting green synthesis aligns with sustainable practices, enhancing corporate social responsibility profiles and appealing to environmentally conscious consumers and investors. Simultaneously, the rise of antibiotic-resistant microbes has fueled the urgent need for novel antimicrobial agents. Green-synthesized nanoparticles, owing to their unique physicochemical properties and diverse biological activities, are emerging as promising candidates for combating microbial infections. The antimicrobial efficacy of these nanoparticles stems from their ability to disrupt microbial cell membranes, inhibit vital enzyme systems, and interfere with microbial replication, thereby offering a multifaceted approach to addressing the growing antimicrobial resistance crisis [20,33].

**Figure 1:**
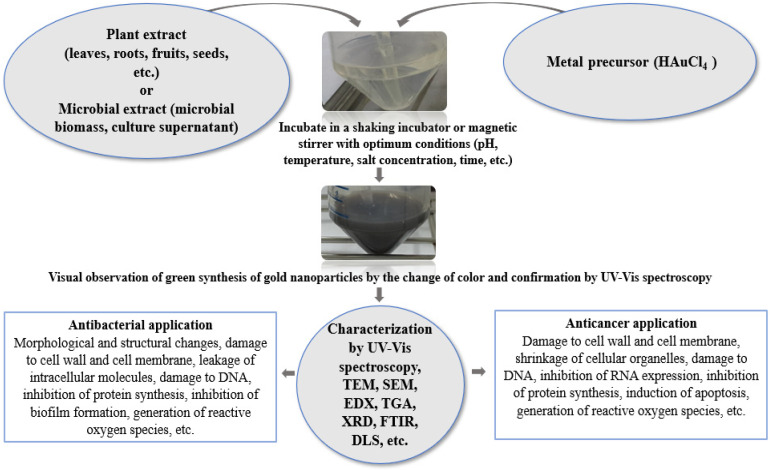
Schematic representation of the green synthesis, characterization and potential antibacterial and anticancer applications of AuNPs.

As we delve deeper into green-synthesized nanoparticles, it becomes increasingly evident that their potential extends far beyond conventional therapeutic approaches. The amalgamation of eco-friendly synthesis methods with nanoparticle technology offers a sustainable path forward, harnessing the power of nature to tackle some of the most pressing challenges in modern medicine. This exploration into the anticancer and antimicrobial applications of green-synthesized nanoparticles promises to reshape the medical landscape, offering hope for more effective, efficient, and environmentally conscious treatment strategies. Among various metal nanoparticles, AuNPs have received a lot of attention due to their wide scope of application in different branches of biomedical science [5,7,34,35]. Many reports suggest applying AuNPs as antibacterial and anticancer agents to control drug-resistant pathogenic microorganisms and cancer cells, respectively [5,7,12,36]. Because of their strong antimicrobial and anticancer efficacies, there is prominent research interest in AuNPs for developing novel, safe, and effective antimicrobial and anticancer agents. Figure 1 shows the schematic representation of the green synthesis, characterization and potential antibacterial and anticancer applications of AuNPs.

This review article provides an overview of the green synthesis of AuNPs using plants and microorganisms and their potential applications against drug-resistant pathogenic bacteria and cancer cells. This review also focuses on the antibacterial and anticancer mechanisms of facile and eco-friendly synthesized AuNPs.

## 2. Plant-Mediated Green Synthesis

The availability of many different plants and their simple and secure use make plant-mediated synthesis of AuNPs an extensively used approach [37]. For green synthesis of bioactive AuNPs, a variety of plant materials, e.g., fruits, roots, flowers, leaves, peels, etc., have been used successfully (Table 1). Many bioactive substances, e.g., proteins, enzymes, amino acids, terpenoids, tannins, saccharides, phenols, and flavonoids, are found in plant extracts [38]. Plant extracts are stable and efficient in the production of bioactive AuNPs [39]. In the last few years, many studies have been performed fon the green synthesis of bioactive AuNPs by utilizing different parts of plants, e.g., leaf, bark, rhizome, seed, fruit, seaweed, latex, flower, peel, and stem, as well as whole plant (Table 1). For example, Sundararajan and Kumari [40] utilized leaf extracts from *Artemisia vulgaris* for rapid and eco-friendly synthesis of bioactive AuNPs. They also examined the antifungal and antimicrobial activity of biosynthesized AuNPs against various pathogenic fungi and bacteria. Nadagouda et al. [41] synthesized AuNPs by utilizing rhizome extracts from *Curcuma longa*. Fruit extract of *Citrus maxima*, citrus (lemon, tangerine, and orange), and *Genipa americana* L., *Lantana camara* were utilized by Majumdar et al. [42], Sujitha and Kannan [43], Sengupta et al. [44], Yu et al. [45], Kumar et al. [46], Dipankar et al. [47], Hidayat et al. [48], and Kumar et al. [49] for the eco-friendly synthesis of AuNPs. The seeds and pulp of *Abelmoschus esculentus*, *Caesalpinia crista*, *Linum usitatissimum*, *Murraya koenigii*, and *Theobromo cacao* were utilized for the green synthesis of AuNPs by Rahaman Mollick et al. [50], Jayaseelan et al. [51], Donga et al. [52], Al-Radadi et al. [53], Ananth et al. [54], and Dwivedi et al. [55]. Peel extracts of different plants, for example *Mangifera indica Linn* (Mango), *Musa paradisiaca*, and *Punica granatum*, have also been investigated for the synthesis of bioactive AuNPs (Table 1).

Optimum synthesis conditions (salt concentration, temperature, and incubation time), size (nm), shape of synthesized AuNPs, and their bioactivity vary greatly depending on the plant or part of the plant used. According to Philip [56], AuNPs of 17 to 20 nm in size were synthesized using the leaf extract of *Mangifera indica* in a two minute reaction. On the other hand, according to Yang et al. [57] AuNPs of 3.26 to 21.68 nm were synthesized using peel extract of *Mangifera indica Linn* (Mango) within 15 mins of reaction. According to Barai et al. [58] stem/bark extracts of *Nerium oleander* produced spherical, hexagonal, triangular, and rod-shaped AuNPs. The leaf extracts of *Abutilon indicum*, *Artocarpus hirsutus*, *Curcumae Kwangsiensis*, *Dracocephalum kotschyi*, *Hibiscus sabdariffa*, *Mangifera indica*, *Mimosa pudica*, *Platycodon grandiflorum*, *Ricinus ommunis*, and *Terminalia arjuna* produced spherical-shaped AuNPs (Table 1). Various physicochemical parameters, such as type of plant, composition of extract, salt concentration, plant extract concentration, extract salt ratio, incubation time, incubation temperature, pH, etc. have a significant impact on the yield, shape, size and stability of the synthesized AuNPs [59,60,61,62]. These physicochemical parameters also influence the efficacy of synthesized AuNPs. For example, increasing the volume of *Carallia brachiata* leaf extract decreased the size of the synthesized AuNPs [61]. Similarly, by increasing the concentration of HAuCl_4_ in the reaction mixture, the synthesis rate, size and shape of *Solidago canadensis* leaf extract mediated AuNPs changed dynamically [62]. According to Singh et al. [63], the size of *Mangifera* peel extract-mediated AuNPs was 6 nm in pH 9 and 18 nm in pH 2. Similarly, the size of *Padina tetrastromatica*-mediated AuNPs was changed with different temperatures [64]. Diksha et al. [65] investigated the optimum conditions for the green synthesis of AuNPs using leaf extract of *Syzygium cumini* and they found that reaction parameters such as salt concentration, plant extract concentration, pH, temperature, and time play an important role in the facile, stable, and rapid synthesis of bioactive AuNPs. Plant-mediated production of AuNPs is likely mediated by the chemistry of reduction and oxidation. According to certain theories, plant extracts include vitamins, proteins, amino acids, organic acids, enzymes, terpenoids, flavonoids, alkaloids, polyphenols, and polysaccharides, which play important roles in the reduction of gold salts as well as acting as capping and stabilizing agents [66,67].

**Table 1:** Plant-mediated green synthesis and potential antibacterial and anticancer applications of AuNPs.

Plant	Used Part	Optimum Synthesis Conditions (Salt Concentration, Temperature, Incubation Time)	Size (nm)	Shape	Applications	Reference
*Clerodendrum trichotomum*	Leaf extract	10.20 g HAuCl_4_ was added to 100 mL of plant extract, incubated at 65 °C, 80 min	19.1 ± 2.2 (Average)	Spherical	Antibacterial application against *Klebsiella pneumoniae* and *Staphylococcus aureus*. Anticancer application against breast cancer cell line (MCF-7)	[2]
Henna	Leaf extract	10 mM HAuCl_4_, room temperature, 30 min	6 ± 2.5 (Average)	Spherical	Antibacterial activity against *Staphylococcus aureus* and *Escherichia coli*	[68]
*Halodule uninervis*	Leaf extract	HAuCl_4_·3H_2_O, 70–80 °C, 30 min	10–50	Spherical	Anticancer activity against human breast cancer cells MDA-MB-231	[69]
*Tangerine*	Peel extract	15 mM HAuCl_4_·3H_2_O, 40 °C, 60 min	26 ± 5 (Average)	Spherical	Antibacterial activity against *Klebsiella pneumoniae*, *Escherichia coli*, and *Pseudomonas aeruginosa*	[70]
*Aconitum violaceum*	Plant extract	1 mM HAuCl_4_, room temperature, 50 min	<100 (Average)	Spherical and triangular	Antibacterial activity against *Lactobacillus acidophilus* and *Escherichia coli*	[71]
*Syzygium cumini*	Leaf extract	HAuCl_4_·3H_2_O, ambient temperature, 24 h	120.5 (Average)	Spherical	Antibacterial application against *Aeromonas hydrophila*, *E. coli*, *Salmonella Typhimurium*, *P. aeruginosa*, *Enterococcus faecium*, *Pediococcus* sp., and *Bacillus cereus*	[72]
*Zingiber officinale*, curcumin	Root extract	HAuCl_4_ solution (0.1 mM) and ginger extract mixture is kept boiling and stirring at 600 rpm until the solution turned purple	20 (Average size)	Spherical and oval	Antimicrobial efficacy against, *E. coli*, *P. aeruginosa* and *S. aureus*	[73]
*Abutilon indicum*	Leaf extract	1 mM HAuCl_4_, room temperature, 2 min	10–20	Spherical	Effective against human colon cancer	[12,74]
*Artemisia vulgaris*	Leaf extracts	1 mM HAuCl_4_ solution, room temperature, 24 h	50–100	Spherical, triangular, and hexagonal	Antibacterial application against *S. aureus*, *S. pyogenes*, *E. coli*, *P. aeruginosa*, anti-fungal activity against *Aspergillus niger*, induced apoptosis in MCF-7 (breast cancer)	[36,40]
*Azadirachta indica*	Leaf extract	100 ppm gold chlorate, the extract and the gold chlorate mixture are boiled till the appearance of wine-red color.	≤121.7	Spherical, hexagonal, and triangular	Anticancer activity on HeLa and MDCK cell	[75]
*Areca catechu*	Nut	30 mL chloroauric acid, 10 mL aqueous nut extract, 4–5 h	22.2	Spherical	Anticancer activity on HeLa	[76]
*Acacia nilotica*	Bark extract	1 mM HAuCl_4_, room temperature, 10 min	10–15	Unshaped, quasispherical	Anticancer activity on hepatic cell, antibacterial activity against *B. subtilis* and *S. aureus*	[77]
*Acorus calamus*	Rhizome extract	2.5 mL of extract, 2.5 mL of 0.001 M chloroauric acid, stir at 240 rpm until the color turns dark brown.	10	Spherical	Antibacterial activity *S. aureus* and *E. coli*	[33,78]
*Artocarpus hirsutus*	Leaf extract	1 mM HAuCl_4_, at 80 °C, about 12 h.	5–40	Spherical	Efficacy against human cancer cell lines (HeLa, RKO and A549).	[34,79]
*Abelmoschus esculentus*	Seed and pulp extract	(1 mM) HAuCl_4_ × H_2_O (95 mL) at room temperature, 1 h	45–75	Spherical, uneven shape	Antibacterial activity against *E. coli*, *P. aeruginosa*, *B. cereus*, and *B. subtilis*. Antifungal activity against *A. niger*, *Puccinia graminis tritci*, and *C. albicans*	[50,51]
*Butea monosperma*	Leaf extract	0.01 M of HAuCl_4_, room temperature, 35 min	20–80	Mainly spherical but with a few rods; irregular and hexagonal	Anticancer activity on B16F10, MCF-7, HNGC2, A549, HUVEC and ECV-304	[12,80]
*Cassia auriculata*	Leaf extract	1 mM auric chloride solution, room temperature, 10 min.	15–25	Spherical, triangular, and hexagonal	Antibacterial efficacy against *Bacillus subtilis*, *K. pneumonia*, *P. aeruginosa*	[81]
*Caesalpinia crista*	Seed extract	1 mM HAuCl_4_, room temperature (25 °C ± 2 °C) for 24 h	15.13	Spherical	Antibacterial efficacy against *B. subtilis*, *S. aureus*, *E. coli*, *K. pneumoniae* Anticancer activity against human cancer cell lines (HeLa, MCF-7),	[52]
Citrus (lemon, tangerine, orange)	Fruit extract	1 mM HAuCl_4_3H_2_O, room temperature	32.3, 43.4, 56.7	Spherical and triangular	Anticancer effect on the growth of HepG2 (liver cancer cell line)	[42,43]
*Citrus maxima*	Fruit extract	(1%, *w*/*v*) HAuCl_4_·4H_2_O, room temperature, 5 min.	15–35	Spherical	Antibacterial efficacy against *Staphylococcus aureus*	[44,45]
*Clitoria ternatea*	Leaf extract	99 mL of 10^−3^ aqueous HAuCl_4_, room temperature for (0 min–24 h)	100	Rod	Antibacterial activity against *E. coli*, *K. pneumoniae*, *S. aureus*, and *S. pyogenes*	[82,83]
*Curcuma longa*	Rhizome extract	0.01 mL HAuCl_4_, room temperature, overnight culture	5–60	Oblong and spherical	Anticancer effect on the lung cancer cells	[41]
*Curcumae Kwangsiensis*	Leaf extracts	(1 mM) HAuCl_4_·H_2_O, 25 °C, 1 h	8–25	Spherical	Anticancer effect on ovarian cancer cell lines i.e., PA-1, SW-626, and SK-OV-3.	[84]
*Dendropanax morbifera*	Leaf extract	1 mM chloroauric acid, 80 °C for 10 min	10–20	Polygonal and hexagonal	Anticancer activity on HaCaT and A549	[85]
*Dracocephalum kotschyi*	Leaf extract	1 mM HAuCl_4_, room temperature for 10 min.	11	Spherical	Anticancer activity on K562 and HeLa	[86]
*Ecklonia cava* (marine brown alga)	Seaweed extract	1 mM chloroauric acid, 80 °C, 10 min	20–50	Spherical and triangular	Anticancer activity on HaCaT, MCF-7	[87,88]
*Genipa americana*	Fruit extract	0.5 mM AuCl_4_ solution and kept at 22–25 °C, 15 min	30.4 ± 14.9	Spherical	Anticancer activity on A-549 and Hela	[46,47]
*Guazuma ulmifolia*	Bark extract	1 mM HAuCl_4_·3H_2_O, room temperature, 1 h	20–25	Spherical	Antibacterial properties against *Staphylococcus aureus* and anticancer activity	[89,90]
*Hibiscus sabdariffa*	Leaf extract	(1 mM) HAuCl_4_ × H_2_O (100 mL), room temperature, 30 min	10–30	Near spherical	Antifungal potentials against *C. krusei*, *C. guilliermondii*, *C. glabrata*, and *C. albicans*, antibacterial effects against *Streptococcus pneumonia*, *Staphylococcus aureus*, *Bacillus subtilis*, *Salmonella typhimurium*, *Escherichia coli*, *and Pseudomonas aeruginosa*. Anticancer activity on U87 and HEK 293	[91,92]
*Justicia glauca*	Leaf extract	1 mM chloroauric acid, room temperature, 10 min	32	Hexagonal and spherical	Antimicrobial effects against *E. coli*, *Streptococcus mutans*, *Micrococcus luteus*, *S. aureus*, *S. cerevisiae*, *Bacillus subtilis*, *L. acidophilus*, *P. aeruginosa*, and *C. albicans*	[93,94]
*Lantana camara*	Fruit extract	0.2 mM AuCl_4_ room temperature (22–25 °C), 72 h	150–300	Triangular	Antibacterial efficacy against *S. aureus*, *E. coli*, *Propionibacterium acnes*, and *P. aeruginosa*	[48,49]
*Linum usitatissimum*	Seed extract	1 mM HAuCl_4_·3H_2_O, room temperature (30 °C), 6 h	3.4–5.7	Spherical and triangular	Anticancer activity on MCF-7, HepG-2, HCT-116	[53]
*Lonicera japonica*	Flower extract	HAuCl_4_ concentration (0.125, 0.5, 1, 1.5, and 2 mM), reaction temperature (40, 50, 60, 70, and 80 °C), reaction time (1, 1.5, 2, 2.5, and 3 min)	8	Triangular and tetrahedral	Anticancer activity on HeLa cells	[95]
*Mangifera indica*	Leaf extract	(5 × 10^−4^ M) HAuCl_4_·3H_2_O, room temperature, 2 min	17–20	Spherical	Anticancer activity on HeLa, MCF-7, Normal fibroblast	[56]
*Mangifera indica* Linn (Mango)	Peel extract	HAuCl_4_ (1.0 mM), incubation at 100 °C, 15 min	3.26–21.68	Quasi-spherical	Anticancer activity on CV-1 and WI-38	[57]
*Mimosa pudica*	Leaf extract	1 mM HAuCl_4_. 3H_2_O, 55 °C, 30 min.	12	Spherical	Anticancer activity on MDA-MB-231, MCF-7 and HMEC	[96]
*Musa paradisiaca*	Peel extract	1 mM HAuCl_4_, 20 min	50	Spherical	Anticancer activity on human lung cancer cells (A549)	[97]
*Murraya koenigii*	Seed extract	1 mM HAuCl_4_, 50 °C, room temperature, 10 min	20–40	Spherical	Antibacterial efficacy against *S. aureus*. *P. aeruginosa* and *Enterococci*	[22,54]
*Nerium oleander*	Stem/bark extract	1 mM HAuCl_4_ room temperature (25 °C ± 2 °C), 24 h	20–40	Spherical, hexagonal, triangular, and rod shaped	Anticancer activity on MCF-7 cell lines	[58]
*Padina gymnospora* (marine Macroalgae)	Leaf extract	1 mM HAuCl_4_, 30 °C, 45 °C, 55 °C, 65 °C, 75 °C, 85 °C and 95 °C, few minutes to hours	14.10 ± 1.5	Spherical	Anticancer activity on HepG2, A549, and 3T3 cell line	[98,99]
*Platycodon grandiflorum*	Leaf extract	HAuCl_4_·3H_2_O (1 mM), (20, 37, and 50 °C), 10 min	15	Spherical	Antibacterial application against *E. coli*, *B. subtilis*	[100]
*Phragmites australis*	Leaf extract	1 mM HAuCl_4_, 85 °C for 1 h	18	Spherical	Anticancer activity on A549 cell line	[101]
*Ricinus ommunis*	Leaf extract	HAuCl_4_ (0.5 mM), 60 °C, 5 min	40–80	Spherical	Antibacterial activity against *S. aureus*, *E. coli*, *P. mirabilis*, *S. flexneri*, *C. albicans*. Anticancer activity on HT29 and SW480 Cancer Cell	[102]
*Pistacia integerrima*	Gall extract	1 mM HAuCl_4_·3H_2_O, 37 °C, 24–72 h	20–200	Grain-like	Antibacterial activity against *K. pneumonia*	[103]
*Sargassum swartzii*	Seaweed	Chloroauric acid (1 mM HAuCl_4_), 60 °C, 5 min	20–60	Spherical and few hexagonal	Anticancer activity on HeLa	[104]
*Terminalia arjuna*	Peel extract	1 mM HAuCl_4_, 80 °C, 15 min	60	Triangular, hexagonal, and pentagonal	Antibacterial activity against *S. aureus*, *P. aeruginosa*, *S. typhimurium*	[105]
*Theobromo cacao*	Seed extract	1 mM HAuCl_4_, (30, 40, 50, 60, and 70 °C), 15 min	150–200	Spherical	Antibacterial activity against A431 cell line	[55,106]
*Zataria multiflora*	Leaf extract	1 mM chloroauric acid (HAuCl_4_), room temperature, few minutes	10–50	Different shapes	Anticancer activity on HeLa and BMSCs cell line	[107]

## 3. Microbe-Mediated Green Synthesis

In recent years, the prospect of utilizing microorganisms for the environment-friendly synthesis of AuNPs has been introduced [108]. It has been demonstrated that microorganisms act as effective biological agents for the simple, economical, and eco-friendly synthesis of AuNPs, avoiding expensive and hazardous chemicals as well as the high energy requirements of physicochemical methods [109]. There are many recent studies on the green synthesis of AuNPs using microorganisms (Table 2). For green synthesis of AuNPs, a variety of microorganisms are frequently used due to their quick growth, simplicity of handling, and ease of cultivation. These include bacteria, yeast, fungi, and algae [110]. Extracellular and intracellular techniques are two ways to use microorganisms for the environment-friendly synthesis of AuNPs [109]. It is yet unknown exactly how microorganisms help in the production of AuNPs. First, microbial enzymes, such as reductase enzyme, convert the metal ions to nanoparticles (NPs) [111]. Then, a variety of microbial extracellular and intracellular biomolecules act as stabilizing and capping agents [112]. Jafari et al. [113] demonstrated extracellular synthesis of AuNPs from *Micrococcus yunnanensis.* The interaction of 1 mM HAuCl_4_, with a bacterial culture supernatant at 30 °C temperature yielded nanoparticles within 24 h of reaction. The size of synthesized AuNPs from FE-TEM analysis was found to range between 15 and 55 nm. Malarkodi et al. [114] also reported the extracellular synthesis of AuNPs using the culture supernatant of a bacterial strain *Klebsiella pneumoniae* within 24 h of reaction at room temperature and found spherical-shaped nanoparticles of 16–50 nm in size.

According to Wen et. al. [115] AuNPs were synthesized through bioreduction of HAuCl_4_ by the culture supernatant of *Bacillus megatherium* D01. *Paraclostridium benzoelyticum*, *Pseudomonas aeruginosa*, and *Streptomyces viridogens* have the ability to produce AuNPs with greater antimicrobial activity [1,116,117]. Shunmugam et al. [118] has also reported intracellular synthesis of AuNPs using *Vibrio alginolyticus*, but found irregular-shaped nanoparticles of a small size in the range of 50–100 nm. AuNPs were also easily and quickly synthesized in an environmentally friendly way using a variety of fungi and algae. For example, the culture supernatant of *Aspergillus niger* was used to produce AuNPs with a size of 5.6 ± 12.8 nm [119,120]. The microbial synthesis of AuNPs is also greatly influenced by different parameters, such as salt concentration, extract salt ratio, incubation time, incubation temperature, pH, etc. Cherian et al. [5], investigated the optimum conditions for stable, facile, and rapid synthesis of AuNPs using the culture supernatant of marine bacterium *Lysinibacillus odysseyi* PBCW2 and they found that various factors such as concentrations of cell-free supernatant and HAuCl_4_, their ratio, pH, incubation temperature, etc., play key roles in the stable and rapid synthesis of AuNPs.

**Table 2:** Microbe-mediated green synthesis and potential antibacterial and anticancer applications of AuNPs.

Microbes Used for Synthesis	Synthesis Method	Optimum Synthesis Conditions (Salt Concentration, Temperature, Incubation Time)	Size (nm)	Shape	Applications	Reference
*Streptomyces* sp. ASM19	Extracellular	1 mM HAuCl_4_, 37 °C for 24 h	6.28 ± 0.78 to 100.2 ± 0.25	Sphere-like form	Antimicrobial activity against *Staphylococcus aureus* and *Escherichia coli*, anticancer potency against liver, colon, breast, and oral carcinoma	[121]
*Streptomyces monashensis* MSK03	Extracellular	1 mM HAuCl_4_, 37 °C for 72 h	7.1–40.0	Spherical	Antibacterial activity against *Pseudomonas aeruginosa* and *Acinetobacter baumannii*	[122]
*Alternaria alternate*	Extracellular	1 mM HAuCl_4_, room temperature for 24 h	12–29	Spherical, triangular, and hexagonal	Antibacterial application against *E. coli* and *S. aureus*	[123]
*Aspergillus flavus*	Extracellular	10 mM HAuCl_4_, 30 °C for 2 min	12	Spherical	Anticancer agent against HepG2 and A549 cell lines	[124]
*Aspergillus clavatus*	Extracellular	1 mM HAuCl_4_, room temperature for 48–72 h	24.4 ± 11	Triangular, spherical, and hexagonal	Antibacterial application against *E. coli* and *S. aureus*	[125]
*Aspergillus foetidus*	Extracellular	1 mM HAuCl_4_, 75 ± 2 °C for 4 h	30–50	Spherical	Anticancer effect on A549	[126,127]
*Aspergillus niger*	Extracellular	1 mM HAuCl_4_, 25 °C for 72 h	5.6 ± 12.8	Spherical	Antibacterial application against *Escherichia coli*, *Pseudomonas aeruginosa* and *Staphylococcus aureus*. Anti larval application against mosquito larvae	[119,120]
*Aspergillus sydowii*	Extracellular	3 mM HAuCl_4_, 27 °C for 72 h	8.7–15.6	Spherical	Antibacterial application against *Staphylococcus aureus*, *Staphylococcus epidermidis*	[128]
*Bacillus flexus*	Extracellular	1 mM aqueous HAuCl_4_, room temperature for 2 h	20	Different shapes (irregular, spherical, and triangular)	Anticancer effect on MCF-7	[129]
*Bacillus megatherium*	Extracellular	10 mg/mL HAuCl_4_, 9 h for 26 °C	1.9 ± 0.8	Spherical	Antibacterial application against *Staphylococcus aureus* and *Bacillus subtilis*	[115]
*Brevibacillus formosus*	Extracellular	1 mM HAuCl_4_, 37 °C for 24 h	5–12	Spherical	Antibacterial application against *Escherichia coli*, *Staphylococcus aureus*	[130]
*Cladosporium* sp.	Extracellular	1 mM Chloroauric acid (HAuCl_4_), 12 h for 37 °C	5–10	Spherical	Anticancer application against MCF-7	[131]
*Caldicellulosiruptor changbaiensis*	Extracellular	500 μM HAuCl_4_·3H_2_O, 12 h for 75 °C	20–60	Spherical	Antibacterial efficacy against *S. aureus* and *E. coli*	[132,133]
*Enterococcus* sp.	Extracellular	1 mM gold chloride, room temperature for 24–48 h	6–13	Spherical	Anticancer application against HepG2 and A549 cell	[134]
*Fusarium solani*	Extracellular	1 mM HAuCl_4_, 28 °C, for 48 h	15–35	Spherical	Anticancer application against HEp2 and Vero cells	[135]
*Fusarium oxysporum*	Extracellular	0.5 mM HAuCl_4_, 30 °C for 24 to 96 h	10–40	Spherical	Anticancer application against ZR-75-1, Daudi and PBMC	[136,137]
*Humicola* spp.	Extracellular	1 mM HAuCl_4_, 50 °C for 96 h	18–24	Spherical	Anticancer application against NIH3T3 and MDA-MB-231	[138,139]
*Micrococcus yunnanensis*	Extracellular	1 mM HAuCl_4_, 30 °C for 24 h	15–55	Spherical	Anticancer application against U87, HT1080, PC12, Caco-2, MCF7, A549. Antibacterial application against *B. subtilis*, *S. typhi*, *Micrococcus luteus*, *E. coli*, *K. pneumoniae*,	[113]
*Pseudomonas aeruginosa*	Extracellular	1 mM HAuCl_4_, 37 °C for 24 h	40 ± 10	Spherical	Antibacterial application against *Enterococcus faecalis*, *S. aureus*, and *E. coli*	[116]
*Penicillium brevicompactum*	Extracellular	1 mM HAuCl_4_, 30 °C for 12–72 h	10–120	Spherical, Triangular, and hexagonal	Anticancer application against C_2_C_12_	[140,141]
*Pleurotus ostreatus*	Extracellular	2.5 mM HAuCl_4_, 37 °C for 24 h	10–30	Spherical and prism-shaped	Anticancer and synergistic antimicrobial activity against *C. albicans*, *P. aeruginosa* and *S. aureus*	[142,143]
*Paracoccus haeundaensis*	Extracellular	2 mM HAuCl_4_·3H_2_O, 25 °C for 48 h	~20	Spherical	Anticancer application against HaCaT A549	[144]
*Rhodopseudomonas capsulata*	Extracellular	1 mM aqueous HAuCl_4_, room temperature for 48 h	10–20	Spherical	Antibacterial application against *E*. *coli* and *S. aureus*	[141,145]
*Streptomyces* sp.	Extracellular	1 mM aqueous HAuCl_4_, 80 °C for 30 min	10–50	Spherical and triangular	Anticancer application against HeLa cell	[146]
*Streptomyces viridogens*	Intracellular	1 mM HAuCl_4_, 28 °C for 120 h	18–20	Spherical	Antibacterial application against *Escherichia coli*, and *S. aureus*	[1,117]
*Streptomyces hygroscopicus*	Extracellular	1 mM HAuCl_4_, 30 °C for 48 h	10–20		Antibacterial application against *E. coli*, *S. typhimurium* and *S. aureus.*	[147]
*Shewanella oneidensis*	Extra cellular	1 mM HAuCl_4_, 30 °C for 48 h	12 ± 5	Spherical	Antibacterial application against *E. coli* and *S. aureus*	[108,148]
*Vibrio alginolyticus*	Intracellular	1 mM aqueous chloroauric acid (HAuCl_4_), 40 °C for 24 h	50–100	Irregular	Anticancer application against HCA-7	[118]

## 4. Characterization of Green Synthesized AuNPs

Various instruments such as ultraviolet-visible spectroscopy (UV-Vis), transmission electron microscopy (TEM), atomic force microscopy (AFM), field emission scanning electron microscopy (FESEM), X-ray diffraction (XRD), energy dispersive spectroscopy (EDS), Fourier-transformed infrared spectroscopy (FTIR), dynamic light scattering (DLS), and zeta potential analysis are commonly used for the characterization of green synthesized AuNPs. These instruments provide valuable information on the physical and chemical properties of AuNPs, including their size, shape, surface charge, crystallinity, and surface functional groups. TEM, FESEM, and AFM have been instrumental in studying the morphology and size of AuNPs. For instance, TEM has been crucial in observing micellar assemblies formed with triblock copolymers acting as soft templates, leading to tiny AuNPs and resulting in micelle–AuNP hybrid assemblies. AFM, on the other hand, has been utilized to optimize the shape and size of AuNPs by employing block copolymers, demonstrating compact aggregates below 10 nm with a mean height. The control of AuNP size at specific locations has been achieved using oriented phases of block copolymers. Spectroscopic methods, specifically UV-visible spectroscopy, have been employed to measure the absorption spectra of individual AuNPs, revealing surface plasmon resonance and indicating the interaction between delocalized electrons on the nanoparticles’ surface and the incident beam. The formation of AuNPs can be followed through the shift of the plasmon in UV-Vis spectroscopy. For example, gold nanoparticles synthesized in the presence of water-soluble micelles in Pluronics exhibited surface plasmon bands at approximately 530–535 nm. Additionally, UV-Vis spectra have been crucial in understanding the inter-particle distances of AuNPs in various polymer systems, providing insights into their growth processes. The crystallinity and purity of synthesized nanoparticles have been examined by XRD analysis. DLS and zeta potential analysis provide information on particle size distribution and surface charge, respectively. Fourier-transformed infrared spectroscopy is an important instrument to investigate the functional groups present on the surfaces of nanoparticles [5,7,149].

**Figure 2:**
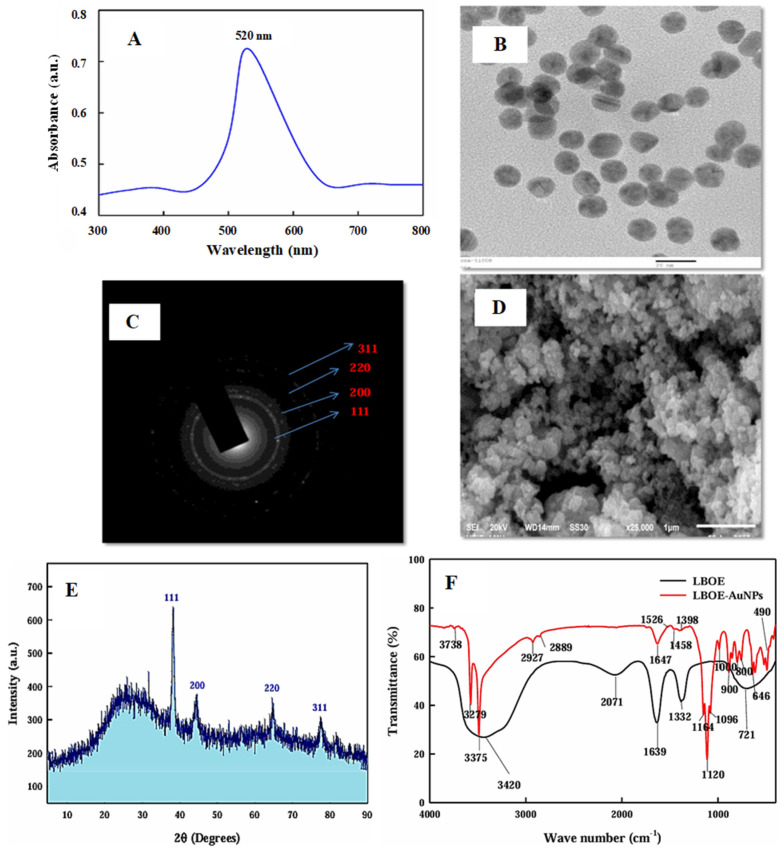
Characterization of LBOE-AuNPs. (**A**) UV–Vis spectrum, (**B**) TEM, (**C**) SAED, (**D**) SEM, (**E**) XRD and (**F**) FTIR spectra of LBOE and LBOE-AuNPs. This figure has been reprinted with permission from ref. [5], copyright 2022, MDPI.

The field of green synthesis of AuNPs has seen significant advancements, as demonstrated by several notable studies. Abdulwahed et al. [150] and Kureshi et al. [151] explored the utilization of plant extracts, specifically from apple (*Malus viridis*) and pepper (*Capsicum annuum*) peels, as both reducing and stabilizing agents in the eco-friendly synthesis of AuNPs. These AuNPs were extensively characterized using techniques such as EDS, FTIR, XRD, AFM, FESEM, UV-Vis, and zeta potential analysis. Their research not only delved into the physical characteristics of the synthesized AuNPs but also explored their biological effects, specifically in inhibiting breast cancer cell line MCF-7, with varying results based on the plant sources used [150]. Cherian et al. [5] used UV-visible spectrophotometry, SEM, TEM, EDX, XRD, TGA, DLS, zeta potential analysis, and FTIR to characterize green-synthesized AuNPs using the marine bacterium *Lysinibacillus odysseyi* PBCW2 and investigate their antioxidant and antibacterial activity. UV-visible spectrophotometry analysis showed a distinct single peak at 520 nm (Figure 2A). TEM analysis revealed the spherical shape of green-synthesized AuNPs (Figure 2B). The SAED pattern showed well-defined diffraction spots in the form of rings, indicating the polycrystalline nature of synthesized AuNPs (Figure 2C). An SEM image depicted small-size nanoparticles with similar shapes and minor-to-negligible aggregations (Figure 2D). XRD analysis confirmed the crystalline structure of synthesized AuNPs with an average size of 29.28 nm (Figure 2E). FTIR analysis revealed various biomolecules present in the bacterial extract and on the surface of synthesized AuNPs responsible for the reducing, capping, and stabilizing of the biosynthesized AuNPs (Figure 2F).

**Figure 3:**
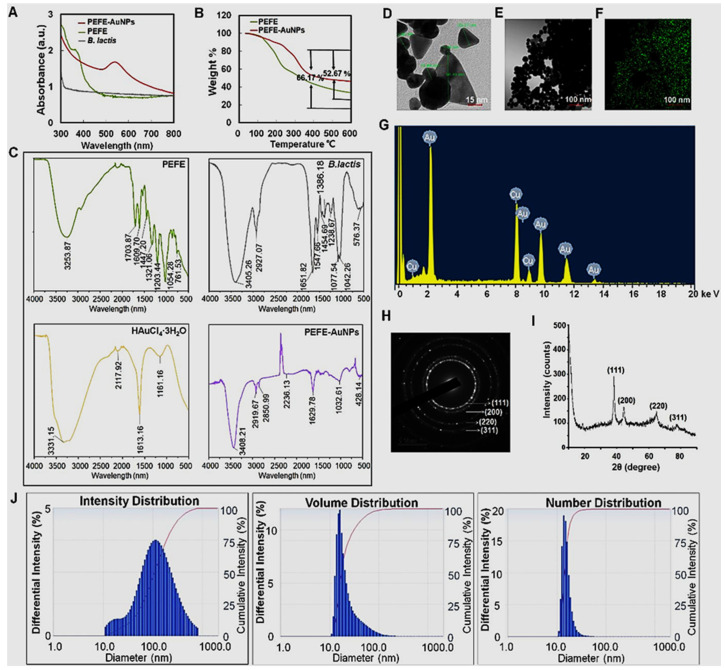
Physiochemical characterization of nanoparticle samples. (**A**) UV–Vis spectrum, (**B**) TGA spectrum, (**C**) FT-IR spectrum, (**D**) FE-TEM image of PEFE-AuNPs, (**E**,**F**) elemental mapping analysis of PEFE-AuNPs, (**G**) EDX analysis of PEFE-AuNPs, (**H**) SAED pattern of PEFE-AuNPs, (**I**) XRD spectrum of PEFE-AuNPs, and (**J**) DLS spectrum of PEFE-AuNPs. This figure has been reprinted with permission from ref. [7], copyright 2021, MDPI.

Kureshi et al. [151] utilized aqueous extracts from the fruit pericarps of *Garcinia indica* (GI) and *Garcinia cambogia* (GC) fruits in the green synthesis of AuNPs. This study focused on characterizing the green-synthesized AuNPs, demonstrating their absorption peak at 541 nm through UV-visible spectroscopy. The AuNPs exhibited spherical and triangular shape morphology, with sizes ranging from 2 to 10 nm. These AuNPs demonstrated significant cytotoxicity against the MCF-7 cancer cell line, with IC50 values of 34.55 µg/mL (GI) and 35.69 µg/mL (GC). Moreover, the AuNPs displayed substantial antioxidant and antibacterial properties, showcasing their potential for various biomedical applications [151]. Wang et al. [7] used UV-visible spectrophotometry, FE-TEM, EDX, SAED, XRD, FTIR, TGA, and DLS, to characterize the green-synthesized AuNPs using *Phyllanthus emblica* fruit extract and *Bifidobacterium animalis* subsp. *lactis* and investigate their anticancer activity against the human gastric carcinoma cell line. UV-visible spectrophotometry analysis showed a distinct single peak at 545 nm, demonstrating the successful synthesis of AuNPs, although there was no peak observed for either the *B. lactis* sample or *Phyllanthus emblica* fruit extract alone, (Figure 3A). Thermogravimetric analysis (TGA) revealed the thermal stability of the green-synthesized AuNPs (Figure 3B). FTIR analysis showed various functional groups present in the *Phyllanthus emblica* fruit extract, bacterial extract, gold salt, and on the surface of synthesized AuNPs (Figure 3C). TEM analysis revealed the circular, triangular, and polygonal nanohybrid shapes of green-synthesized AuNPs with 5–60 nm size (Figure 3D,E). EDX analysis indicated that Au was the main element in the synthesized AuNPs (Figure 3F,G). The SAED pattern showed well-defined diffraction spots, metallic characteristics, and circular structure, indicating the crystalline nature of synthesized AuNPs (Figure 3H). XRD analysis also confirmed the crystalline structure of synthesized AuNPs (Figure 3I). DLS analysis revealed the average hydrodynamic size of synthesized nanoparticles based on number, volume, and intensity (Figure 3J).

## 5. Antibacterial Applications and Mechanisms of Green-Synthesized AuNPs

**Figure 4:**
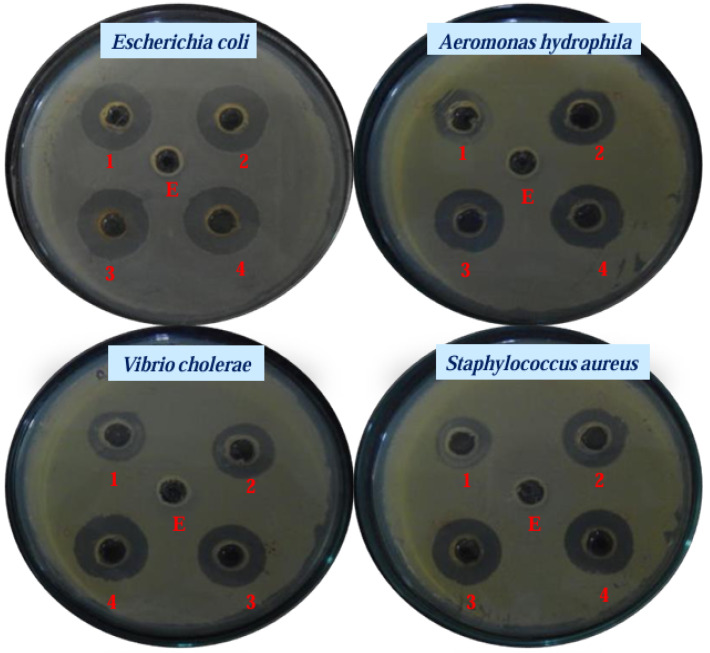
Antibacterial activity of LBOE (E) and LBOE-AuNPs at (1) 20, (2) 60, (3) 80, and (4) 100 µg/mL based on the agar well diffusion assay. This figure has been reprinted with permission from ref. [5], copyright 2022, MDPI.

The growing menace of antibacterial resistance has underscored the urgency for innovative strategies to combat infectious diseases. Green-synthesized AuNPs have emerged as promising candidates due to their potent antimicrobial activity. AuNPs have been extensively studied for their ability to inhibit the growth of pathogenic microorganisms, including drug-resistant pathogenic bacteria [33,119,123]. Zheng [152] conducted a study highlighting the efficacy of ultrasmall gold nanoclusters (AuNCs) with dimensions less than 2 nm in manifesting a broad-spectrum antimicrobial effect against both Gram-positive and Gram-negative bacteria. The diminutive size of these AuNCs facilitated enhanced interactions with bacterial cells, resulting in metabolic perturbations and heightened generation of reactive oxygen species, thereby inducing bacterial cell death [152]. Contrastingly, Zhang et al. [153] proposed an alternative perspective, suggesting that the antimicrobial activity of AuNPs could be ascribed to co-existing chemical factors, including gold ions and surface coating agents, rather than the intrinsic properties of the nanoparticles themselves. This nuanced interpretation underscores the importance of considering multiple facets when elucidating the antibacterial mechanisms of AuNPs. Moreover, investigations by Lokina et al. [154] delved into the synthesis of AuNPs utilizing *Punica Granatum* fruit extract. The resulting AuNPs exhibited noteworthy antibacterial efficacy against a spectrum of microorganisms, providing a novel and natural source for the fabrication of antimicrobial nanomaterials [154]. Additionally, the multifunctionality of AuNPs was demonstrated by their capacity to augment the bactericidal effects of antibiotics when employed as carriers or delivery vehicles. This finding, as suggested by several studies, opens avenues for synergistic approaches in antimicrobial therapy, wherein AuNPs act as potent adjuncts to conventional antibiotics, enhancing their efficacy [152,154]. The amalgamation of findings from these studies elucidates the diverse mechanisms and applications of AuNPs in the realm of antimicrobial activity, encompassing both size-dependent interactions with bacterial cells and the potential contributions of co-existing chemical constituents. The synthesis methodologies, such as the implementation of natural extracts, further broaden the scope of utilizing AuNPs for combating microbial infections. Cherian et al. [5] reported the green synthesis of AuNPs using the culture supernatant of marine bacterium *Lysinibacillus odysseyi* PBCW2 and evaluated their antimicrobial activity against several pathogenic bacteria such as *Vibrio cholera* MTCC 3905, *Escherichia coli* serotype 0115, *Aeromonas hydrophila* IDH1585, and *Staphylococcus aureus*. They found that *Lysinibacillus odysseyi* PBCW2-mediated synthesized AuNPs strongly suppressed the growth of these pathogenic bacteria and showed a strong zone of inhibition (Figure 4).

Zhao [155] contributes to the elucidation of the antibacterial mechanisms of gold nanoparticles (AuNPs) by demonstrating that pyrimidine-capped gold nanoparticles can disrupt bacterial cell membranes. This disruption leads to the leakage of cytoplasmic contents and subsequent inhibition of protein synthesis. The study sheds light on the specific molecular interactions between AuNPs and bacterial cells, underscoring the importance of membrane disruption as a key aspect of AuNPs’ antibacterial activity [155]. In a different vein, Lee and Lee [156] emphasized the synergistic antibacterial activity of AuNPs, although the explicit mechanism underlying this synergy is not explicitly discussed. The study hints at complex interactions between AuNPs and bacterial cells, suggesting that the antibacterial effects may result from a combination of multiple factors. The exploration of this synergistic antibacterial activity opens avenues for further investigation into the nuanced interplay between AuNPs and bacterial pathogens [156]. Timoszyk and Grochowalska [60] delved into the antibacterial activity of AuNPs functionalized with natural compounds derived from plants. The study underscores the significance of natural coating in influencing interactions with bacterial cell walls, providing insights into the role of surface modifications in enhancing antibacterial efficacy. This approach highlights the potential of using plant-derived compounds to functionalize AuNPs for optimized antibacterial applications [60]. Mahdi and Parveen [157] focused on the biosynthesis of AuNPs using black lemon extract and investigated their antibacterial activity against both Gram-positive and Gram-negative bacteria. The study showcases a bioinspired approach to AuNP synthesis and emphasizes the broad-spectrum antibacterial efficacy of the resulting nanoparticles. This work contributes to the expanding repertoire of environmentally friendly methods for AuNP synthesis with potent antibacterial applications [157].

Dasari et al. [158] explored the antibacterial activity of Au(I) and Au(III) against different bacteria, revealing their toxicity to both nonpathogenic and multidrug-resistant strains. Buffer composition significantly affected bacterial growth inhibition, highlighting the importance of experimental conditions when studying AuNP toxicity. Centrifugation-resuspension successfully removed residual Au(III) ions from AuNPs, making them non-toxic. Wang et al. [159] developed a photosensitizer-loaded hybrid nanostructure with high antibacterial efficiency. AuNPs on the polymer surface amplified the photosensitizer’s effects, leading to increased reactive oxygen species (ROS) generation. The nanostructure exhibited significant antibacterial efficacy against *Escherichia coli* in the presence of lectin protein, providing an innovative approach for controlled antibacterial assays. Khan et al. [160] employed biogenic synthesis using *Acer pentapomicum* leaf extract to produce AuNPs. These nanoparticles exhibited spherical morphology and demonstrated antibacterial, antifungal, and antioxidant activities, showcasing their potential in various applications. Mahmoud et al. [161] developed a library of gold nanorods (GNRs) with different coatings, investigating their penetration into skin layers. Cholesterol-PEG-coated GNRs showed preferential accumulation in the upper skin layers, demonstrating antibacterial activity against *Staphylococcus aureus*. These findings open new avenues for utilizing gold-based nanoscale systems in skin disease therapy [161]. These studies collectively emphasize the diverse strategies and conditions impacting the antibacterial properties of AuNPs, showcasing their potential in various biomedical applications.

**Figure 5:**
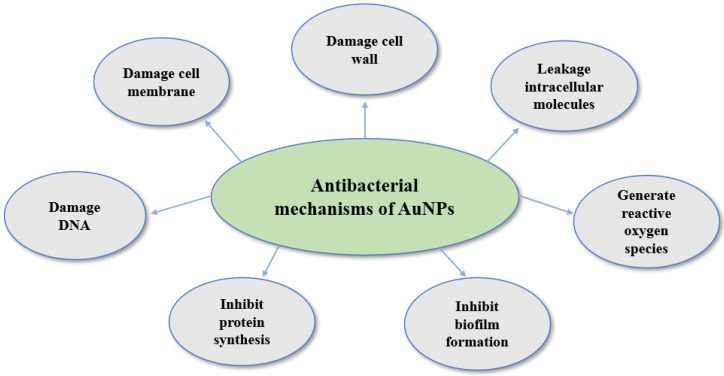
Proposed antibacterial mechanisms of AuNPs.

In summary, these studies collectively underscore the multifaceted nature of AuNPs regarding antibacterial activity. The mechanisms include membrane disruption, inhibition of protein synthesis, interactions with bacterial cell walls, and potentially synergistic effects. This diverse range of antibacterial mechanisms highlights the versatility of AuNPs and their potential in developing effective strategies against bacterial infections. One of the key mechanisms underlying the antibacterial action of AuNPs involves their ability to disrupt the bacterial cell membrane integrity. These nanoparticles can easily penetrate the bacterial cell wall, leading to membrane damage and subsequent cell death. The small size and large surface area of AuNPs enhance their interactions with bacterial cells, allowing for efficient binding and disruption of the cell membrane. Additionally, AuNPs can generate reactive oxygen species (ROS) upon interaction with bacterial cells. ROS, such as superoxide radicals and hydrogen peroxide, cause oxidative stress and damage to cellular components, leading to bacterial cell death. Moreover, AuNPs can interfere with bacterial enzymes and proteins, disrupting essential cellular processes. Another intriguing aspect of the antibacterial mechanism of AuNPs is their ability to inhibit biofilm formation. Biofilms, which are communities of bacteria encased in a protective extracellular matrix, are notoriously resistant to conventional antibiotics. AuNPs have shown the potential to prevent biofilm formation and disrupt pre-formed biofilms, making them valuable candidates for combating persistent bacterial infections. Furthermore, AuNPs can be functionalized with various antibacterial agents, enhancing their efficacy and specificity against specific bacterial strains. The surface modification of AuNPs with molecules like antibiotics, peptides, or polymers allows for targeted delivery of antibacterial agents to specific bacterial cells while minimizing damage to healthy cells, thus reducing side effects. The versatility of AuNPs in terms of size, shape, and surface chemistry further contributes to their wide-ranging antibacterial applications. Researchers continue to explore innovative methods to optimize AuNPs’ antibacterial properties and develop novel strategies for combating antibiotic-resistant bacteria. As the understanding of AuNPs’ mechanisms of action deepens, their potential to revolutionize antibacterial therapy becomes increasingly evident, offering hope for more effective and targeted treatments against infectious diseases. Figure 5 shows the proposed antibacterial mechanisms of AuNPs.

## 6. Anticancer Applications and Mechanisms of Green Synthesized AuNPs

The fight against cancer is a major global challenge, driving relentless efforts to discover safe and effective anticancer drugs. Green-synthesized AuNPs could be a potential game-changer in this case. Many studies have reported the strong anticancer efficacy of green-synthesized AuNPs against different cancer cell lines [22,34,42,126,162]. Barai and colleagues reported that AuNPs synthesized from *Nerium oleander* bark extract induced an anti-breast cancer effect in MCF-7 cell lines [58]. Also, grapefruit extract-derived AuNPs showed anticancer properties in HeLa cell lines. Eight green botanical extracts of different plant components were used to create AuNPs, and their anticancer properties were examined against MCF 7 (breast cancer) cell lines [163]. *Cirsium japonicum* extract was used in the biological synthesis of AuNPs, which displayed anticancer impacts on stomach cancer (AGS cell lines) [164]. According to research, AuNPs from plant extracts of *Catharanthus roseus* and *Carica papaya* may be able to treat acute lymphocytic leukemia, breast cancer, Hodgkin’s disease, leukemia, lymphomas, neuroblastoma, soft tissue sarcomas, and multiple myeloma [165]. Similarly, *Citrus macroptera* fruit extract was used to produce AuNPs, which were then tested for anticancer action on three distinct malignant cell lines: MDA-MB 468 (breast cancer cell), tA 549 (alveolar basal epithelial cells), and HepG2 (liver cancer cell line) [166]. Furthermore, extracts of *Taxus baccata*, *Marsdenia tenacissima*, *Lonicera japonica*, *Abies spectabilis*, *Gymnema sylvestre*, *Panax notoginseng*, and *Sasa borealis* were used for the green synthesis of AuNPs and the synthesized AuNPs exhibited potential anticancer effects in the following cancerous cell types: the HT29 cell line [167], PANIC-1 cells [168], AGS cell lines [169], lung cancer A549 cells [170], cervix cancer (HeLa) cells [95], bladder cancer T24 cell lines [171], cervical cancer HeLa cells [172], breast adenocarcinoma (MCF-7) cancer cells [173], and various cancerous cells like breast (MCF-7), cervical (HeLa), and ovarian (Caov-4) [174]. In a 2023 study, G Tan et al. reported garlic and onion extract-mediated green synthesis of AuNPs and their anticancer activity in MCF-7 cells [175]. In addition, *Halymenia pseudofloresii* extract, bovine serum albumin, *Hypnea valentiae* seaweed, *Sonneratia alba* fruits, jellyfish nematocyst venom protein, and *Nothapodytes foetida* leaf extract were used for the green synthesis of AuNPs and displayed potential anticancer effects in lung cancer cells [176], cervix cancer cells (HeLa) [177], lung cancer A549 cells [178], lung cancer cell line A549 [179], breast cancer MCF-7 cells [180], cervix cancer cell line KB-3-1 [181], and lung cancer A549 cell lines [182].

**Figure 6:**
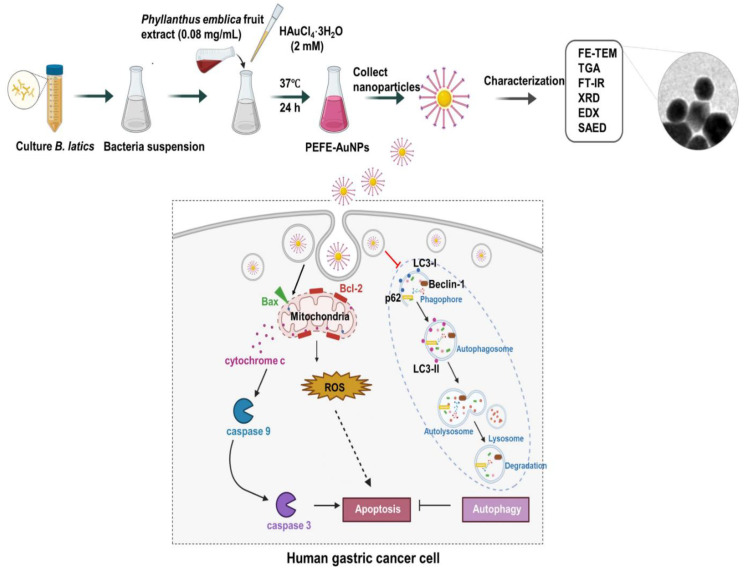
Green synthesis of gold nanoparticles using *Phyllanthus emblica* fruit extract and *Bifidobacterium animalis* subsp. *lactis* and their anticancer effect on human gastric cancer cells. This figure has been reprinted with permission from ref. [7], copyright 2022, MDPI.

The antitumor effect of AuNPs against liver and cancer cells was investigated using marine microbe *Entercoccus* sp.-mediated green-synthesized AuNPs [134]. AuNPs synthesized by the bacteria *Streptomyces griseus* displayed anti-breast (MCF-7 cell lines) and colon (HCT-116 cell lines) cancer impacts [183]. *Exiguobacterium aestuarii*-mediated green-synthesized AuNPs showed apoptosis activity in human breast cancer MCF-7 cells [184]. AuNPs synthesized with red algae (*Halymenia pseudofloresii*) trigger apoptosis in lung cancer cells by ROS formation [176]. *Commiphora wightii* plant conjugated with AuNPs and fungus (*Cladosporium sp.*) showed an anticancer effect against breast (MCF-7 cell lines) cancer [131]. In MCF10A and MCF7 cancer cells, AuNPs were conjugated with *Microalga dunaliella* (which is halotolerant algae) to show anticancer action using green technology [185]. AuNPs were synthesized by *Sargassum glaucescens* (brown seaweed), and their activity checked in the breast (MDA-MB-231), cervical (HeLa), leukemia (CEM-ss), and liver (HepG2) cancerous cell lines [186]. In another report, extract of *Sargassum incisifolium* was employed to make AuNPs and confirm their toxicity in cancer cells (MCF-7 and HT-29), which does not appear to be harmful to the environment [187]. Wang et al. [7] reported the green synthesis of AuNPs using both the fruit extract of *Phyllanthus emblica* and culture supernatant of *Bifidobacterium animalis* subsp. *lactis* and investigated their anticancer activity against human gastric carcinoma cell lines. They found that *Phyllanthus emblica* fruit extract and *Bifidobacterium animalis* subsp. *Lactis*-mediated green-synthesized AuNPs strongly suppressed the growth of the human gastric adenocarcinoma cell line (AGS). They concluded that the anticancer activity of synthesized AuNPs against gastric cancer cells was associated with the induction of apoptosis through inhibition of autophagy, downregulation of LC3-II/LC3-I and Beclin-1 expression, and upregulation of p62 expression in AGS cells [7]. Figure 6 shows the green synthesis of gold nanoparticles using *Phyllanthus emblica* fruit extract and *Bifidobacterium animalis* subsp. *lactis* and their anticancer effect on human gastric cancer cells.

The broad surface area of AuNPs makes them highly capable of mixing, enabling them to be conjugated to various compounds, including anticancer agents and other biomolecules [163]. Since most capillaries have pores that range in size from 6 to 12 nm, the size of the nanoparticles makes them simpler for tumor cell membranes to penetrate. Therefore, bigger nanoparticles will facilitate simpler extravasation. Additionally, tumor arteries typically include pores that range in size from 40 to 200 nm [188]. Therefore, the efficacy of nanoparticles can be increased by conjugating these nanoparticles with other anticancer agents. For instance, anti-VEGF protein conjugated with AuNPs is being revealed to enhance the apoptosis and cell development in CLL cells in comparison to their antibody [189]. AuNPs achieve maximum efficacy, minimal side effects, and reduce damage to normal (noncancerous) cells [164]. AuNPs show synergistic cancer therapeutic [190] and cytotoxic effects against cancerous cells depending on the nanoparticle dose [191]. Conjugation of bioactive AuNPs with natural ligands could be a good idea for the development of novel, safe, and effective anticancer agents. Several studies have reported on the conjugation of bioactive AuNPs with various ligands including polyethylene glycol, folic acid, hyaluronic acid, naturally occurring proteins, and peptides, etc. [192,193]. Conjugation of these ligands with bioactive AuNPs aids in targeted delivery, minimizes cytotoxic effects on normal cells and increases anticancer efficacies [192,193].

The anticancer effect of AuNPs is attributed to a complex and poorly understood mechanism. Several molecular mechanisms of anticancer activity of green-synthesized AuNPs have been reported. These mechanisms include induction of apoptosis, mitochondrial dysfunction, inhibition of angiogenesis, and generation of reactive oxygen species, etc. [194]. AuNPs are used for component transporters and exhibit an anticancer effect [191]. Researchers have reported that the interaction of AuNPs with cells is occurs in different ways, such as cellular internalization of AuNPs [195]. AuNP properties of the surface are significant factors in cell uptake. Cancer and normal cell membranes contain negatively charged substances such as lipids (particularly phosphate), and AuNPs with positive charges are taken up and internalized by these membranes with opposite charges [196,197]. Endocytosis is another method for AuNPs to enter cells, as demonstrated by a study in which small AuNPs were endocytosed and aggregation was observed in HeLa cells [198]. The AuNPs exhibited cytotoxic action through ROS formation [199], damage to DNA [191], and triggering of caspase cascade, resulting in apoptotic and mitochondrial abnormalities [164,199]. According to reports, ROS generation, mitochondrial malfunction, and apoptosis triggered by caspase are caused by AuNPs mediated by *phyllanthus emblica* extract [7].

**Figure 7:**
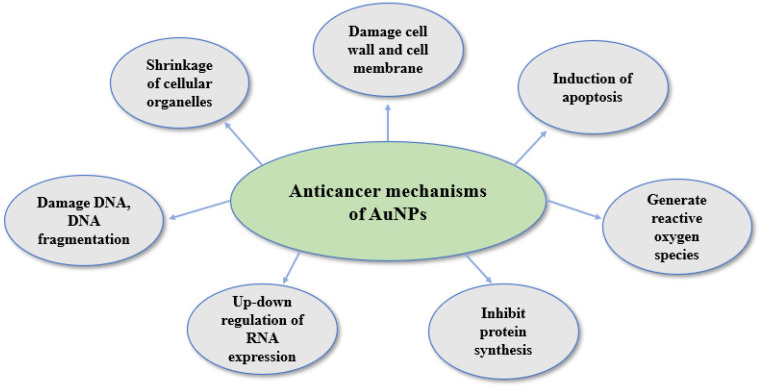
Proposed mechanisms of action of green-synthesized AuNPs against cancer cells.

*Rhus chinensis* plant extract-conjugated AuNPs have shown effects on multiple cancerous cells, exhibiting cytotoxic action through DNA damage [200]. *P. hexandrum* AuNPs produced comparable effects on HeLa cells [191]. Apoptosis induced by activation of caspase cascades, which includes caspase cascades 3, 8, and 9, and cell cycle arrest in the G2/M phase was also seen [201]. *Moringa oleifera*-conjugated AuNPs in lung cancer A549 cells were also observed to exhibit caspase-mediated apoptosis, as evidenced by an increase in caspase 3/7 and 9 activity, a substantial decrease in ATP levels, and a noticeable rise in Bax and p53 levels of proteins [202]. Figure 7 shows the proposed mechanisms of action of green-synthesized AuNPs against cancer cells.

## 7. Conclusions and Future Prospects

The emergence of antibacterial and anticancer drug resistance severely threatens global public health. The growing menace of antibacterial and anticancer drug resistance has underscored the urgency for innovative strategies to combat cancer and infectious diseases caused by pathogenic microorganisms. Hence, it is essential to develop novel, safe, and effective antimicrobial and anticancer agents. Bioactive AuNPs could be promising agents to solve this problem. Recently, many studies have been conducted on the green synthesis of AuNPs using either plants or microorganisms and investigated their potential application against drug-resistant pathogenic bacteria as well as cancer cells. The present review provides an overview of the green synthesis of AuNPs using plants and microorganisms and their potential applications against drug-resistant pathogenic bacteria and cancer cells. Moreover, this review focuses on the antibacterial and anticancer mechanisms of facile and eco-friendly synthesized AuNPs. Green synthesis of AuNPs using plants and microbes is a facile, non-toxic, cost-effective, and eco-friendly method. Plant extracts contain various bioactive compounds, and microbial extracts also contain numerous metabolites that act as reducing, capping, and stabilizing agents during the synthesis of AuNPs. The antibacterial activity of green-synthesized AuNPs is attributed to several mechanisms, including morphological and structural changes, damage to the cell wall and cell membrane, leakage of intracellular molecules, damage to DNA, inhibition of protein synthesis, inhibition of biofilm formation, generation of reactive oxygen species, etc. Similarly, the anticancer activity of green-synthesized AuNPs is attributed to several mechanisms, including damage to the cell wall and cell membrane, shrinkage of cellular organelles, damage to DNA, up–down regulation of RNA expression, inhibition of protein synthesis, induction of apoptosis, generation of reactive oxygen species, etc. Although many reports have already been published on the green synthesis of AuNPs for the development of antimicrobial and anticancer agents, some challenges remain. The challenges include large-scale production, long-term stability, biocompatibility, targeted delivery to minimize cytotoxic effects on normal cells, and the absence of clear clinical trial data. Several points might be considered for the future synthesis of AuNPs to address these challenges. First of all, the selection of a plant or microbial extract that contains antibacterial or anticancer-active biomolecules. If the plant or microbial extract contains antibacterial or anticancer-active biomolecules, the efficacy of green-synthesized AuNPs could be greatly increased. Second, the optimization of various synthesis parameters, such as salt concentration, plant or microbial extract concentration, extract salt ratio, incubation time, incubation temperature, pH, etc., is very important for high, stable, and rapid synthesis of AuNPs. Third, the conjugation of green-synthesized antibacterial or anticancer-active AuNPs with antibacterial or anticancer drugs or other antibacterial or anticancer-active biomolecules could be a great approach for the development of novel and effective antibacterial and anticancer drugs. Conjugation of bioactive AuNPs with ligands could be a good idea for targeted delivery to minimize cytotoxic effects on normal cells. Fourth, the potential adverse effects of green-synthesized antibacterial and anticancer-active AuNPs on human health and the environment should be studied. It is very important to investigate the biocompatibility and biosafety of synthesized AuNPs using a proper in vitro and in vivo screening model system. Finally, clinical trials should be conducted for the development of novel, safe, and effective antibacterial or anticancer-active AuNPs.

## References

[1] Abirami B., Akshata V., Radhakrishnan M., Namitha R., Govindaraju K., Gopikrishnan V., Manigundan K. (2023). Characterization of biosynthesized gold nanoparticles from *Streptomyces misionensis* PYA9 with biomedical and environmental applications. Int. J. Agric. Technol..

[2] Shakoor A., Ferdous U.T., Khan S.A., Gulzar M.M. (2025). Green Synthesis of Gold Nanoparticles Using *Clerodendrum trichotomum* Thunberg for Antibacterial and Anticancer Applications. Int. J. Nanomed..

[3] Varshan G.A., Namasivayam S.K.R. (2025). A Critical Review on Sustainable Formulation of Anti-quorum Sensing Compounds Using Nanotechnology Principles Against *Candida albicans*. BioNanoScience.

[4] Huq M.A., Ashrafudoulla M., Rahman M.M., Balusamy S.R., Akter S. (2022). Green synthesis and potential antibacterial applications of bioactive silver nanoparticles: A review. Polymers.

[5] Cherian T., Maity D., Rajendra Kumar R.T., Balasubramani G., Ragavendran C., Yalla S., Mohanraju R., Peijnenburg W.J. (2022). Green chemistry based gold nanoparticles synthesis using the marine bacterium *Lysinibacillus odysseyi* PBCW2 and their multitudinous activities. Nanomaterials.

[6] Gour A., Jain N.K. (2019). Advances in green synthesis of nanoparticles. Artif. Cells Nanomed. Biotechnol..

[7] Wang R., Xu X., Puja A.M., Perumalsamy H., Balusamy S.R., Kim H., Kim Y.-J. (2021). Gold nanoparticles prepared with *Phyllanthus emblica* fruit extract and *Bifidobacterium animalis* subsp. lactis can induce apoptosis via mitochondrial impairment with inhibition of autophagy in the human gastric carcinoma cell line AGS. Nanomaterials.

[8] Jadoun S., Arif R., Jangid N.K., Meena R.K. (2021). Green synthesis of nanoparticles using plant extracts: A review. Environ. Chem. Lett..

[9] Dikshit P.K., Kumar J., Das A.K., Sadhu S., Sharma S., Singh S., Gupta P.K., Kim B.S. (2021). Green Synthesis of Metallic Nanoparticles: Applications and Limitations. Catalysts.

[10] Huq M.A., Apu M.A.I., Ashrafudoulla M., Rahman M.M., Parvez M.A.K., Balusamy S.R., Akter S., Rahman M.S. (2023). Bioactive ZnO nanoparticles: Biosynthesis, characterization and potential antimicrobial applications. Pharmaceutics.

[11] Deepak P., Amutha V., Kamaraj C., Balasubramani G., Aiswarya D., Perumal P. (2019). Chemical and green synthesis of nanoparticles and their efficacy on cancer cells. Green Synthesis, Characterization and Applications of Nanoparticles.

[12] Huang X., Devi S., Bordiga M., Brennan C.S., Xu B. (2023). Phenolic compounds mediated biosynthesis of gold nanoparticles and evaluation of their bioactivities: A review. Int. J. Food Sci. Technol..

[13] Taha R.H. (2022). Green synthesis of silver and gold nanoparticles and their potential applications as therapeutics in cancer therapy; a review. Inorg. Chem. Commun..

[14] Anselmo A.C., Mitragotri S. (2019). Nanoparticles in the clinic: An update. Bioeng. Transl. Med..

[15] Huq M.A., Akter S. (2021). Biosynthesis, characterization and antibacterial application of novel silver nanoparticles against drug resistant pathogenic *Klebsiella pneumoniae* and *Salmonella* enteritidis. Molecules.

[16] Lampé I., Beke D., Biri S., Csarnovics I., Csik A., Dombrádi Z., Hajdu P., Hegedűs V., Rácz R., Varga I. (2019). Investigation of silver nanoparticles on titanium surface created by ion implantation technology. Int. J. Nanomed..

[17] Akter S., Lee S.-Y., Siddiqi M.Z., Balusamy S.R., Ashrafudoulla M., Rupa E.J., Huq M.A. (2020). Eco-friendly synthesis of silver nanoparticles by *Terrabacter humi* sp. nov. and their antibacterial application against antibiotic-resistant pathogens. Int. J. Mol. Sci..

[18] Wang X., Lee S.-Y., Akter S., Huq M.A. (2022). Probiotic-mediated biosynthesis of silver nanoparticles and their antibacterial applications against pathogenic strains of *Escherichia coli* O157:H7. Polymers.

[19] Ying S., Guan Z., Ofoegbu P.C., Clubb P., Rico C., He F., Hong J. (2022). Green synthesis of nanoparticles: Current developments and limitations. Environ. Technol. Innov..

[20] Huq M.A., Akter S. (2021). Characterization and genome analysis of *Arthrobacter bangladeshi* sp. nov., applied for the green synthesis of silver nanoparticles and their antibacterial efficacy against drug-resistant human pathogens. Pharmaceutics.

[21] Pal G., Rai P., Pandey A. (2019). Green synthesis of nanoparticles: A greener approach for a cleaner future. Green Synthesis, Characterization and Applications of Nanoparticles.

[22] Bhagat D.S., Gurnule W.B., Bumbrah G.S., Koinkar P., Chawla P.A. (2023). Recent advances in biomedical applications of biogenic nanomaterials. Curr. Pharm. Biotechnol..

[23] Khatami M., Sharifi I., Nobre M.A., Zafarnia N., Aflatoonian M.R. (2018). Waste-grass-mediated green synthesis of silver nanoparticles and evaluation of their anticancer, antifungal and antibacterial activity. Green. Chem. Lett. Rev..

[24] Yusuf A., Almotairy A.R.Z., Henidi H., Alshehri O.Y., Aldughaim M.S. (2023). Nanoparticles as drug delivery systems: A review of the implication of nanoparticles’ physicochemical properties on responses in biological systems. Polymers.

[25] Akter S., Huq M.A. (2020). Biologically rapid synthesis of silver nanoparticles by *Sphingobium* sp. MAH-11T and their antibacterial activity and mechanisms investigation against drug-resistant pathogenic microbes. Artif. Cells Nanomed. Biotechnol..

[26] Mayegowda S.B., Sarma G., Gadilingappa M.N., Alghamdi S., Aslam A., Refaat B., Almehmadi M., Allahyani M., Alsaiari A.A., Aljuaid A. (2023). Green-synthesized nanoparticles and their therapeutic applications: A review. Green. Process. Synth..

[27] Sánchez-López E., Gomes D., Esteruelas G., Bonilla L., Lopez-Machado A.L., Galindo R., Cano A., Espina M., Ettcheto M., Camins A. (2020). Metal-based nanoparticles as antimicrobial agents: An overview. Nanomaterials.

[28] Bhattacharjee R., Negi A., Bhattacharya B., Dey T., Mitra P., Preetam S., Kumar L., Kar S., Das S.S., Iqbal D. (2023). Nanotheranostics to target antibiotic-resistant bacteria: Strategies and applications. OpenNano.

[29] Huq M.A. (2020). Biogenic silver nanoparticles synthesized by *Lysinibacillus xylanilyticus* MAHUQ-40 to control antibiotic-resistant human pathogens *Vibrio parahaemolyticus* and *Salmonella Typhimurium*. Front. Bioeng. Biotechnol..

[30] Wypij M., Jędrzejewski T., Trzcińska-Wencel J., Ostrowski M., Rai M., Golińska P. (2021). Green synthesized silver nanoparticles: Antibacterial and anticancer activities, biocompatibility, and analyses of surface-attached proteins. Front. Microbiol..

[31] Elgamouz A., Idriss H., Nassab C., Bihi A., Bajou K., Hasan K., Abu Haija M., Patole S.P. (2020). Green synthesis, characterization, antimicrobial, anti-cancer, and optimization of colorimetric sensing of hydrogen peroxide of algae extract capped silver nanoparticles. Nanomaterials.

[32] Mohamad Sukri S.N.A., Shameli K., Teow S.-Y., Chew J., Ooi L.-T., Lee-Kiun Soon M., Ismail N.A., Moeini H. (2023). Enhanced antibacterial and anticancer activities of plant extract mediated green synthesized zinc oxide-silver nanoparticles. Front. Microbiol..

[33] Qamar M., Ahmad N., Ismail T., Esatbeyoglu T., Akhtar S., Mubarak M.S. (2023). Medicinal Uses, Phytochemistry, and Pharmacological Properties of Acorus calamus. Aquatic Medicinal Plants.

[34] Muddapur U.M., Alshehri S., Ghoneim M.M., Mahnashi M.H., Alshahrani M.A., Khan A.A., Iqubal S.S., Bahafi A., More S.S., Shaikh I.A. (2022). Plant-based synthesis of gold nanoparticles and theranostic applications: A review. Molecules.

[35] Huq M.A., Ashrafudoulla M., Parvez M.A.K., Balusamy S.R., Rahman M.M., Kim J.H., Akter S. (2022). Chitosan-coated polymeric silver and gold nanoparticles: Biosynthesis, characterization and potential antibacterial applications: A review. Polymers.

[36] Rehman A.U., Tabassum A., Aftab A., Zahid N., Jamal A., Sajini A.A., Gul A., Ahmad B. (2023). Artemisia vulgaris reduced and stabilized titanium oxide nanoparticles for anti-microbial, anti-fungal and anti-cancer activity. Appl. Nanosci..

[37] Rani N., Singh P., Kumar S., Kumar P., Bhankar V., Kumar K. (2023). Plant-mediated synthesis of nanoparticles and their applications: A review. Mater. Res. Bull..

[38] Dhaka A., Mali S.C., Sharma S., Trivedi R. (2023). A review on biological synthesis of silver nanoparticles and their potential applications. Results Chem..

[39] Kumar N., Devra V. (2021). Plant extract mediated synthesis of transition metal nanoparticles: A review. Int. J. Res. Appl. Sci. Eng. Technol..

[40] Sundararajan B., Kumari B.R. (2017). Novel synthesis of gold nanoparticles using *Artemisia vulgaris* L. leaf extract and their efficacy of larvicidal activity against dengue fever vector *Aedes aegypti* L.. J. Trace Elem. Med. Biol..

[41] Nadagouda M.N., Iyanna N., Lalley J., Han C., Dionysiou D.D., Varma R.S. (2014). Synthesis of silver and gold nanoparticles using antioxidants from blackberry, blueberry, pomegranate, and turmeric extracts. ACS Sustain. Chem. Eng..

[42] Majumdar M., Khan S.A., Biswas S.C., Roy D.N., Panja A.S., Misra T.K. (2020). In vitro and in silico investigation of anti-biofilm activity of Citrus macroptera fruit extract mediated silver nanoparticles. J. Mol. Liq..

[43] Sujitha M.V., Kannan S. (2013). Green synthesis of gold nanoparticles using Citrus fruits (*Citrus limon*, *Citrus reticulata* and *Citrus sinensis*) aqueous extract and its characterization. Spectrochim. Acta Part A Mol. Biomol. Spectrosc..

[44] Sengupta S., Saha M., Ghosh N.R., Bhattacharya M., Chatterjee S., Ghosh R. (2023). Green synthesis of gold nano-conjugates using commonly used citrus species and evaluation of its In-vitro antibacterial efficacy against *Staphylococcus aureus*: A comparative study. Int. J. Herb. Med..

[45] Yu J., Xu D., Guan H.N., Wang C., Huang L.K., Chi D.F. (2016). Facile one-step green synthesis of gold nanoparticles using Citrus maxima aqueous extracts and its catalytic activity. Mater. Lett..

[46] Kumar B., Smita K., Cumbal L., Camacho J., Hernández-Gallegos E., de Guadalupe Chávez-López M., Grijalva M., Andrade K. (2016). One pot phytosynthesis of gold nanoparticles using *Genipa americana* fruit extract and its biological applications. Mater. Sci. Eng. C.

[47] Dipankar C., Murugan S. (2012). The green synthesis, characterization and evaluation of the biological activities of silver nanoparticles synthesized from *Iresine herbstii* leaf aqueous extracts. Colloids Surf. B Biointerfaces.

[48] Hidayat H., Purwiandono G., Tohari T., Nugroho B.H., Jauhari M.H., Widyaputra S.B., Fatimah I. (2022). Antibacterial and photocatalytic activity of visible-light-induced synthesized gold nanoparticles by using *Lantana camara* flower extract. Green. Process. Synth..

[49] Kumar B., Smita K., Cumbal L., Debut A. (2017). Extracellular biofabrication of gold nanoparticles by using *Lantana camara* berry extract. Inorg. Nano-Met. Chem..

[50] Rahaman Mollick M.M., Bhowmick B., Mondal D., Maity D., Rana D., Dash S.K., Chattopadhyay S., Roy S., Sarkar J., Acharya K. (2014). Anticancer (in vitro) and antimicrobial effect of gold nanoparticles synthesized using *Abelmoschus esculentus* (L.) pulp extract via a green route. RSC Adv..

[51] Jayaseelan C., Ramkumar R., Rahuman A.A., Perumal P. (2013). Green synthesis of gold nanoparticles using seed aqueous extract of *Abelmoschus esculentus* and its antifungal activity. Ind. Crops Prod..

[52] Donga S., Bhadu G.R., Chanda S., Morrow G.L. (2022). Facile, Low Cost and Eco-Friendly Synthesis of Gold Nanoparticles Using Caesalpinia Crista Seed Extract and Evaluation of their Antimicrobial, Antioxidant and Anticancer Efficacies. Applications of Gold Nanoparticles.

[53] Al-Radadi N.S. (2021). Green biosynthesis of flaxseed gold nanoparticles (Au-NPs) as potent anti-cancer agent against breast cancer cells. J. Saudi Chem. Soc..

[54] Ananth S., Induja M., Thangamathi P., Prabha D., Vinotha K. (2018). In vitro antibacterial activity of biogenic gold nanoparticles from *Murraya koenigii* seed extract against pathogens associated with traumatic wound infections. Int. J. Fauna Biol. Stud..

[55] Dwivedi K., Mandal A.K., Afzal O., Altamimi A.S.A., Sahoo A., Alossaimi M.A., Almalki W.H., Alzahrani A., Barkat M.A., Almeleebia T.M. (2023). Emergence of nano-based formulations for effective delivery of flavonoids against topical infectious disorders. Gels.

[56] Philip D. (2010). Rapid green synthesis of spherical gold nanoparticles using *Mangifera indica* leaf. Spectrochim. Acta Part A Mol. Biomol. Spectrosc..

[57] Yang N., WeiHong L., Hao L. (2014). Biosynthesis of Au nanoparticles using agricultural waste mango peel extract and its in vitro cytotoxic effect on two normal cells. Mater. Lett..

[58] Barai A.C., Paul K., Dey A., Manna S., Roy S., Bag B.G., Mukhopadhyay C. (2018). Green synthesis of *Nerium oleander*-conjugated gold nanoparticles and study of its in vitro anticancer activity on MCF-7 cell lines and catalytic activity. Nano Converg..

[59] Rónavári A., Igaz N., Adamecz D.I., Szerencsés B., Molnar C., Kónya Z., Pfeiffer I., Kiricsi M. (2021). Green silver and gold nanoparticles: Biological synthesis approaches and potentials for biomedical applications. Molecules.

[60] Timoszyk A., Grochowalska R. (2022). Mechanism and antibacterial activity of gold nanoparticles (AuNPs) functionalized with natural compounds from plants. Pharmaceutics.

[61] Ahmad Kuthi N., Chandren S., Basar N., Jamil M.S.S. (2022). Biosynthesis of gold nanoisotrops using *Carallia brachiata* leaf extract and their catalytic application in the reduction of 4-nitrophenol. Front. Chem..

[62] Mariychuk R., Grulova D., Grishchenko L.M., Linnik R.P., Lisnyak V.V. (2020). Green synthesis of non-spherical gold nanoparticles using *Solidago canadensis* L. extract. Appl. Nanosci..

[63] Singh P., Pandit S., Garnæs J., Tunjic S., Mokkapati V.R., Sultan A., Thygesen A., Mackevica A., Mateiu R.V., Daugaard A.E. (2018). Green synthesis of gold and silver nanoparticles from *Cannabis sativa* (industrial hemp) and their capacity for biofilm inhibition. Int. J. Nanomed..

[64] Princy K., Gopinath A. (2018). Optimization of physicochemical parameters in the biofabrication of gold nanoparticles using marine macroalgae *Padina tetrastromatica* and its catalytic efficacy in the degradation of organic dyes. J. Nanostructure Chem..

[65] Diksha D., Gupta S.K., Gupta P., Banerjee U.C., Kalita D., Gupta S. (2023). Antibacterial potential of gold nanoparticles synthesized from leaf extract of *Syzygium cumini* against multidrug-resistant urinary tract pathogens. Cureus.

[66] Mobaraki F., Momeni M., Barghbani M., Far B.F., Hosseinian S., Hosseini S.M. (2022). Extract-mediated biosynthesis and characterization of gold nanoparticles: Exploring their protective effect against cyclophosphamide-induced oxidative stress in rat testis. J. Drug Deliv. Sci. Technol..

[67] Can M. (2020). Green gold nanoparticles from plant-derived materials: An overview of the reaction synthesis types, conditions, and applications. Rev. Chem. Eng..

[68] Mohammadzadeh M., Labbaf S., Kermanpur A. (2025). Eco-friendly synthesis of gold nanoparticles using henna extract: Toward medical applications. Mater. Lett..

[69] Wehbe N., Mesmar J.E., El Kurdi R., Al-Sawalmih A., Badran A., Patra D., Baydoun E. (2025). Halodule uninervis extract facilitates the green synthesis of gold nanoparticles with anticancer activity. Sci. Rep..

[70] Ghoreishi S.M., Mortazavi-Derazkola S. (2025). Eco-friendly synthesis of gold nanoparticles via tangerine peel extract: Unveiling their multifaceted biological and catalytic potentials. Heliyon.

[71] Ahmad S., Ahmad S., Xu Q., Khan I., Cao X., Yang R., Yan H. (2024). Green synthesis of gold and silver nanoparticles using crude extract of *Aconitum violaceum* and evaluation of their antibacterial, antioxidant and photocatalytic activities. Front. Bioeng. Biotechnol..

[72] Das G., Shin H.-S., Lim K.-J., Patra J.K. (2024). Bio-inspired synthesis of gold nanoparticles using leaf extract of *Jamun* and research on its biomedical potential. Int. J. Nanomed..

[73] Kalantari H., Turner R.J. (2024). Structural and antimicrobial properties of synthesized gold nanoparticles using biological and chemical approaches. Front. Chem..

[74] Mata R., Nakkala J.R., Sadras S.R. (2015). Biogenic silver nanoparticles from Abutilon indicum: Their antioxidant, antibacterial and cytotoxic effects in vitro. Colloids Surf. B Biointerfaces.

[75] Dharmatti R., Phadke C., Mewada A., Thakur M., Pandey S., Sharon M. (2014). Biogenic gold nano-triangles: Cargos for anticancer drug delivery. Mater. Sci. Eng. C.

[76] Rajan A., Vilas V., Philip D. (2015). Studies on catalytic, antioxidant, antibacterial and anticancer activities of biogenic gold nanoparticles. J. Mol. Liq..

[77] Dogara A.M., Hama H.A., Ozdemir M. (2024). Biological evaluation of *Acacia nilotica* (L.) Willd. ex Delile: A systematic review. Adv. Tradit. Med..

[78] Ganesan R., Prabu H.G. (2019). Synthesis of gold nanoparticles using herbal *Acorus calamus* rhizome extract and coating on cotton fabric for antibacterial and UV blocking applications. Arab. J. Chem..

[79] Vijayashree I., Niranjana P., Prabhu G., Sureshbabu V., Manjanna J. (2017). Conjugation of Au nanoparticles with chlorambucil for improved anticancer activity. J. Clust. Sci..

[80] Patra S., Mukherjee S., Barui A.K., Ganguly A., Sreedhar B., Patra C.R. (2015). Green synthesis, characterization of gold and silver nanoparticles and their potential application for cancer therapeutics. Mater. Sci. Eng. C.

[81] Kumar V.G., Gokavarapu S.D., Rajeswari A., Dhas T.S., Karthick V., Kapadia Z., Shrestha T., Barathy I., Roy A., Sinha S. (2011). Facile green synthesis of gold nanoparticles using leaf extract of antidiabetic potent *Cassia auriculata*. Colloids Surf. B Biointerfaces.

[82] Fatimah I., Citradewi P.W., Yahya A., Nugroho B.H., Hidayat H., Purwiandono G., Sagadevan S., Ghazali S.A.I.S.M., Ibrahim S. (2021). Biosynthesized gold nanoparticles-doped hydroxyapatite as antibacterial and antioxidant nanocomposite. Mater. Res. Express.

[83] Vanaraj S., Jabastin J., Sathiskumar S., Preethi K. (2017). Production and characterization of bio-AuNPs to induce synergistic effect against multidrug resistant bacterial biofilm. J. Clust. Sci..

[84] Chen J., Li Y., Fang G., Cao Z., Shang Y., Alfarraj S., Alharbi S.A., Li J., Yang S., Duan X. (2021). Green synthesis, characterization, cytotoxicity, antioxidant, and anti-human ovarian cancer activities of Curcumae kwangsiensis leaf aqueous extract green-synthesized gold nanoparticles. Arab. J. Chem..

[85] Zuhrotun A., Oktaviani D.J., Hasanah A.N. (2023). Biosynthesis of gold and silver nanoparticles using phytochemical compounds. Molecules.

[86] Dorosti N., Jamshidi F. (2016). Plant-mediated gold nanoparticles by Dracocephalum kotschyi as anticholinesterase agent: Synthesis, characterization, and evaluation of anticancer and antibacterial activity. J. Appl. Biomed..

[87] Yang S., Li D., Liu W., Chen X. (2023). Polysaccharides from marine biological resources and their anticancer activity on breast cancer. RSC Med. Chem..

[88] Venkatesan J., Manivasagan P., Kim S.-K., Kirthi A.V., Marimuthu S., Rahuman A.A. (2014). Marine algae-mediated synthesis of gold nanoparticles using a novel *Ecklonia cava*. Bioprocess. Biosyst. Eng..

[89] Patel A. (2023). Metal nanoparticles produced by plants with antibacterial properties against *Staphylococcus aureus*. Braz. J. Biol..

[90] Karthika V., Arumugam A., Gopinath K., Kaleeswarran P., Govindarajan M., Alharbi N.S., Kadaikunnan S., Khaled J.M., Benelli G. (2017). Guazuma ulmifolia bark-synthesized Ag, Au and Ag/Au alloy nanoparticles: Photocatalytic potential, DNA/protein interactions, anticancer activity and toxicity against 14 species of microbial pathogens. J. Photochem. Photobiol. B Biol..

[91] Zangeneh M.M., Zangeneh A. (2020). Novel green synthesis of Hibiscus sabdariffa flower extract conjugated gold nanoparticles with excellent anti-acute myeloid leukemia effect in comparison to daunorubicin in a leukemic rodent model. Appl. Organomet. Chem..

[92] Mishra P., Ray S., Sinha S., Das B., Khan M.I., Behera S.K., Yun S.-I., Tripathy S.K., Mishra A. (2016). Facile bio-synthesis of gold nanoparticles by using extract of *Hibiscus sabdariffa* and evaluation of its cytotoxicity against U87 glioblastoma cells under hyperglycemic condition. Biochem. Eng. J..

[93] Balkrishna A., Rohela A., Kumar A., Mishra S., Arya V., Kala V., Thakur N., Thakur N., Kumari A., Khan N. (2023). Elucidating the Role of Plant Extracts Mediated Gold Nanoparticles as Smart Antimicrobials: Two-Way Attack. J. Nanomater..

[94] Karuppiah C., Palanisamy S., Chen S.-M., Emmanuel R., Muthupandi K., Prakash P. (2015). Green synthesis of gold nanoparticles and its application for the trace level determination of painter’s colic. RSC Adv..

[95] Patil M.P., Bayaraa E., Subedi P., Piad L.L.A., Tarte N.H., Kim G.-D. (2019). Biogenic synthesis, characterization of gold nanoparticles using *Lonicera japonica* and their anticancer activity on HeLa cells. J. Drug Deliv. Sci. Technol..

[96] KS U.S., Govindaraju K., Prabhu D., Arulvasu C., Karthick V., Changmai N. (2016). Anti-proliferative effect of biogenic gold nanoparticles against breast cancer cell lines (MDA-MB-231 & MCF-7). Appl. Surf. Sci..

[97] Vijayakumar S., Vaseeharan B., Malaikozhundan B., Gopi N., Ekambaram P., Pachaiappan R., Velusamy P., Murugan K., Benelli G., Kumar R.S. (2017). Therapeutic effects of gold nanoparticles synthesized using *Musa paradisiaca* peel extract against multiple antibiotic resistant *Enterococcus faecalis* biofilms and human lung cancer cells (A549). Microb. Pathog..

[98] Chaudhary V., Chowdhury R., Thukral P., Pathania D., Saklani S., Rustagi S., Gautam A., Mishra Y.K., Singh P., Kaushik A. (2023). Biogenic green metal nano systems as efficient anti-cancer agents. Environ. Res..

[99] Singh M., Saurav K., Majouga A., Kumari M., Kumar M., Manikandan S., Kumaraguru A. (2015). The cytotoxicity and cellular stress by temperature-fabricated polyshaped gold nanoparticles using marine macroalgae, *Padina gymnospora*. Biotechnol. Appl. Biochem..

[100] Anbu P., Gopinath S.C., Jayanthi S. (2020). Synthesis of gold nanoparticles using *Platycodon grandiflorum* extract and its antipathogenic activity under optimal conditions. Nanomater. Nanotechnol..

[101] Oladipo A.O., Iku S.I., Ntwasa M., Nkambule T.T., Mamba B.B., Msagati T.A. (2020). Doxorubicin conjugated hydrophilic AuPt bimetallic nanoparticles fabricated from *Phragmites australis*: Characterization and cytotoxic activity against human cancer cells. J. Drug Deliv. Sci. Technol..

[102] Soto K.M., Luzardo-Ocampo I., López-Romero J.M., Mendoza S., Loarca-Piña G., Rivera-Muñoz E.M., Manzano-Ramírez A. (2022). Gold nanoparticles synthesized with common mullein (*Verbascum thapsus*) and castor bean (*Ricinus communis*) ethanolic extracts displayed antiproliferative effects and induced caspase 3 activity in human HT29 and SW480 cancer cells. Pharmaceutics.

[103] Islam N.U., Jalil K., Shahid M., Muhammad N., Rauf A. (2019). Pistacia integerrima gall extract mediated green synthesis of gold nanoparticles and their biological activities. Arab. J. Chem..

[104] Dhas T.S., Kumar V.G., Karthick V., Govindaraju K., Narayana T.S. (2014). Biosynthesis of gold nanoparticles using *Sargassum swartzii* and its cytotoxicity effect on HeLa cells. Spectrochim. Acta Part A Mol. Biomol. Spectrosc..

[105] Suganthy N., Sri Ramkumar V., Pugazhendhi A., Benelli G., Archunan G. (2018). Biogenic synthesis of gold nanoparticles from *Terminalia arjuna* bark extract: Assessment of safety aspects and neuroprotective potential via antioxidant, anticholinesterase, and antiamyloidogenic effects. Environ. Sci. Pollut. Res..

[106] Fazal S., Jayasree A., Sasidharan S., Koyakutty M., Nair S.V., Menon D. (2014). Green synthesis of anisotropic gold nanoparticles for photothermal therapy of cancer. ACS Appl. Mater. Interfaces.

[107] Baharara J., Ramezani T., Divsalar A., Mousavi M., Seyedarabi A. (2016). Induction of apoptosis by green synthesized gold nanoparticles through activation of caspase-3 and 9 in human cervical cancer cells. Avicenna J. Med. Biotechnol..

[108] Nadeem M., Pervez L., Khan A.M., Burton R.A., Ullah S., Nadhman A., Celli J. (2023). Microbial-mediated synthesis of gold nanoparticles—Current insights and future vistas. Gold. Bull..

[109] Singh N.A., Narang J., Garg D., Jain V., Payasi D., Suleman S., Swami R.K. (2023). Nanoparticles synthesis via microorganisms and their prospective applications in agriculture. Plant Nano Biol..

[110] Varimadugu A., CVS A., Kansoth A.N., Mokkapati V., Koodalingam D., Salla S. (2023). Microbial synthesis of gold nanoparticles. Microbial Processes for Synthesizing Nanomaterials.

[111] Menon K.G., Reddy K.V., Ranjit P., Sree N.R.S. (2023). Microbial enzymes in nanoparticle synthesis. Microbial Processes for Synthesizing Nanomaterials.

[112] Patil T., Gambhir R., Vibhute A., Tiwari A.P. (2023). Gold nanoparticles: Synthesis methods, functionalization and biological applications. J. Clust. Sci..

[113] Jafari M., Rokhbakhsh-Zamin F., Shakibaie M., Moshafi M.H., Ameri A., Rahimi H.R., Forootanfar H. (2018). Cytotoxic and antibacterial activities of biologically synthesized gold nanoparticles assisted by *Micrococcus yunnanensis* strain J2. Biocatal. Agric. Biotechnol..

[114] Malarkodi C., Rajeshkumar S., Vanaja M., Paulkumar K., Gnanajobitha G., Annadurai G. (2013). Eco-friendly synthesis and characterization of gold nanoparticles using* Klebsiella pneumoniae*. J. Nanostructure Chem..

[115] Wen L., Lin Z., Gu P., Zhou J., Yao B., Chen G., Fu J. (2009). Extracellular biosynthesis of monodispersed gold nanoparticles by a SAM capping route. J. Nanoparticle Res..

[116] Husseiny M., Abd El-Aziz M., Badr Y., Mahmoud M. (2007). Biosynthesis of gold nanoparticles using *Pseudomonas aeruginosa*. Spectrochim. Acta Part A Mol. Biomol. Spectrosc..

[117] Balagurunathan R., Radhakrishnan M., Rajendran R.B., Velmurugan D. (2011). Biosynthesis of gold nanoparticles by actinomycete *Streptomyces viridogens* strain HM10. Indian J. Biochem. Biophys..

[118] Shunmugam R., Balusamy S.R., Kumar V., Menon S., Lakshmi T., Perumalsamy H. (2021). Biosynthesis of gold nanoparticles using marine microbe (*Vibrio alginolyticus*) and its anticancer and antioxidant analysis. J. King Saud. Univ. Sci..

[119] Alzamily I.A., Al-Shibly M.K., Ali Z.A.H. (2023). Biosynthesis of Gold Nanoparticles by Aspergillus Niger And Their Role Against Pathogenic Bacteria. Clin. Med. Health Res. J..

[120] Soni N., Prakash S. (2012). Synthesis of gold nanoparticles by the fungus *Aspergillus niger* and its efficacy against mosquito larvae. Rep. Parasitol..

[121] Aati S., Aati H.Y., Hamed A.A., El-Shamy S., Aati S.H., Abdelmohsen U.R., Bringmann G., Taha M.N., Hassan H.M., Bahr H.S. (2025). Gold nanoparticles synthesized from soil-derived *Streptomyces* sp. ASM19: Characterization, antimicrobial, anticancer potency targeted G2/M phase cell-cycle arrest, and in silico studies. RSC Adv..

[122] Kerdtoob S., Chanthasena P., Rosyidah A.l., Limphirat W., Penkhrue W., Ganta P., Srisakvarangkool W., Yasawong M., Nantapong N. (2024). Streptomyces monashensis MSK03-mediated synthesis of gold nanoparticles: Characterization and antibacterial activity. RSC Adv..

[123] Kamal M., Abdel-Raouf N., Sonbol H., Abdel-Tawab H., Abdelhameed M.S., Hammouda O., Elsayed K.N. (2022). In vitro assessment of antimicrobial, anti-inflammatory, and schistolarvicidal activity of macroalgae-based gold nanoparticles. Front. Mar. Sci..

[124] Abu-Tahon M.A., Ghareib M., Abdallah W.E. (2020). Environmentally benign rapid biosynthesis of extracellular gold nanoparticles using *Aspergillus flavus* and their cytotoxic and catalytic activities. Process Biochem..

[125] Verma V.C., Singh S.K., Solanki R., Prakash S. (2011). Biofabrication of anisotropic gold nanotriangles using extract of endophytic *Aspergillus clavatus* as a dual functional reductant and stabilizer. Nanoscale Res. Lett..

[126] Brandelli A., Veras F.F., Rai M., Golinska P. (2023). Biosynthesis of Gold Nanoparticles by Fungi. Mycosynthesis of Nanomaterials: Perspectives and Challenges.

[127] Roy S., Das T.K., Maiti G.P., Basu U. (2016). Microbial biosynthesis of nontoxic gold nanoparticles. Mater. Sci. Eng. B.

[128] Vala A.K. (2015). Exploration on green synthesis of gold nanoparticles by a marine-derived fungus *Aspergillus sydowii*. Environ. Prog. Sustain. Energy.

[129] Murugan M., Anthony K.J.P., Jeyaraj M., Rathinam N.K., Gurunathan S. (2014). Biofabrication of gold nanoparticles and its biocompatibility in human breast adenocarcinoma cells (MCF-7). J. Ind. Eng. Chem..

[130] Srinath B., Namratha K., Byrappa K. (2017). Eco-friendly synthesis of gold nanoparticles by gold mine bacteria *Brevibacillus* formosus and their antibacterial and biocompatible studies. IOSR J. Pharm..

[131] Munawer U., Raghavendra V.B., Ningaraju S., Krishna K.L., Ghosh A.R., Melappa G., Pugazhendhi A. (2020). Biofabrication of gold nanoparticles mediated by the endophytic *Cladosporium* species: Photodegradation, in vitro anticancer activity and in vivo antitumor studies. Int. J. Pharm..

[132] Bing W., Sun H., Wang F., Song Y., Ren J. (2018). Hydrogen-producing hyperthermophilic bacteria synthesized size-controllable fine gold nanoparticles with excellence for eradicating biofilm and antibacterial applications. J. Mater. Chem. B.

[133] Facal Marina P., Kaul L., Mischer N., Richter K. (2022). Metal-Based Nanoparticles for Biofilm Treatment and Infection Control: From Basic Research to Clinical Translation. Antibiofilm Strateg. Curr. Future Appl. Prev. Control Eradicate Biofilms.

[134] Rajeshkumar S. (2016). Anticancer activity of eco-friendly gold nanoparticles against lung and liver cancer cells. J. Genet. Eng. Biotechnol..

[135] Clarance P., Luvankar B., Sales J., Khusro A., Agastian P., Tack J.-C., Al Khulaifi M.M., Al-Shwaiman H.A., Elgorban A.M., Syed A. (2020). Green synthesis and characterization of gold nanoparticles using endophytic fungi *Fusarium solani* and its in-vitro anticancer and biomedical applications. Saudi J. Biol. Sci..

[136] Jampílek J., Kráľová K. (2023). Mycosynthesis of metal-based nanoparticles and their perspectives in agri-food and veterinary/medical applications. Fungal Cell Factories for Sustainable Nanomaterials Productions and Agricultural Applications.

[137] Naimi-Shamel N., Pourali P., Dolatabadi S. (2019). Green synthesis of gold nanoparticles using *Fusarium oxysporum* and antibacterial activity of its tetracycline conjugant. J. Mycol. Medicale.

[138] Syed A., Al Saedi M.H., Bahkali A.H., Elgorgan A.M., Kharat M., Pai K., Pichtel J., Ahmad A. (2022). αAu2S nanoparticles: Fungal-mediated synthesis, structural characterization and bioassay. Green. Chem. Lett. Rev..

[139] Santra T.S., Tseng F.-G., Barik T.K. (2014). Green biosynthesis of gold nanoparticles and biomedical applications. Am. J. Nano Res. Appl..

[140] Mishra A., Tripathy S.K., Wahab R., Jeong S.-H., Hwang I., Yang Y.-B., Kim Y.-S., Shin H.-S., Yun S.-I. (2011). Microbial synthesis of gold nanoparticles using the fungus *Penicillium brevicompactum* and their cytotoxic effects against mouse mayo blast cancer C_2_C_12_ cells. Appl. Microbiol. Biotechnol..

[141] Rajesh N., Lakshmi L.V., Shankar A.S., Basha P.O. (2023). Biosynthesis, Characterization and Applications of Gold Nanoparticles. Microbial Processes for Synthesizing Nanomaterials.

[142] Rai S.N., Mishra D., Singh P., Singh M.P., Vamanu E., Petre A. (2023). Biosynthesis and bioapplications of nanomaterials from mushroom products. Curr. Pharm. Des..

[143] El Domany E.B., Essam T.M., Ahmed A.E., Farghali A.A. (2018). Biosynthesis physico-chemical optimization of gold nanoparticles as anti-cancer and synergetic antimicrobial activity using *Pleurotus ostreatus* fungus. J. Appl. Pharm. Sci..

[144] Patil M.P., Kang M.-j., Niyonizigiye I., Singh A., Kim J.-O., Seo Y.B., Kim G.-D. (2019). Extracellular synthesis of gold nanoparticles using the marine bacterium *Paracoccus haeundaensis* BC74171T and evaluation of their antioxidant activity and antiproliferative effect on normal and cancer cell lines. Colloids Surf. B Biointerfaces.

[145] He S., Guo Z., Zhang Y., Zhang S., Wang J., Gu N. (2007). Biosynthesis of gold nanoparticles using the bacteria *Rhodopseudomonas capsulata*. Mater. Lett..

[146] Manivasagan P., Oh J. (2015). Production of a novel fucoidanase for the green synthesis of gold nanoparticles by *Streptomyces* sp. and its cytotoxic effect on HeLa cells. Mar. Drugs.

[147] Sadhasivam S., Shanmugam P., Veerapandian M., Subbiah R., Yun K. (2012). Biogenic synthesis of multidimensional gold nanoparticles assisted by *Streptomyces hygroscopicus* and its electrochemical and antibacterial properties. Biometals.

[148] Suresh A.K., Pelletier D.A., Wang W., Broich M.L., Moon J.-W., Gu B., Allison D.P., Joy D.C., Phelps T.J., Doktycz M.J. (2011). Biofabrication of discrete spherical gold nanoparticles using the metal-reducing bacterium *Shewanella oneidensis*. Acta Biomater..

[149] Huq M.A., Khan A.A., Alshehri J.M., Rahman M.S., Balusamy S.R., Akter S. (2023). Bacterial mediated green synthesis of silver nanoparticles and their antibacterial and antifungal activities against drug-resistant pathogens. R. Soc. Open Sci..

[150] Abdulwahed S.H., Alias M., MohammedHasan Z. (2021). Green Synthesis and Characterization of Gold Nanoparticles from *Malus viridisand Capsicum annuum* as AnticancerAgent. J. Phys. Conf. Ser..

[151] Kureshi A.A., Vaghela H.M., Kumar S., Singh R., Kumari P. (2020). Green synthesis of gold nanoparticles mediated by *Garcinia* fruits and their biological applications. Pharm. Sci..

[152] Zheng K., Setyawati M.I., Leong D.T., Xie J. (2017). Antimicrobial gold nanoclusters. ACS Nano.

[153] Zhang Y., Shareena Dasari T.P., Deng H., Yu H. (2015). Antimicrobial activity of gold nanoparticles and ionic gold. J. Environ. Sci. Health Part C.

[154] Lokina S., Suresh R., Giribabu K., Stephen A., Sundaram R.L., Narayanan V. (2014). Spectroscopic investigations, antimicrobial, and cytotoxic activity of green synthesized gold nanoparticles. Spectrochim. Acta Part A Mol. Biomol. Spectrosc..

[155] Zhao Y., Tian Y., Cui Y., Liu W., Ma W., Jiang X. (2010). Small molecule-capped gold nanoparticles as potent antibacterial agents that target gram-negative bacteria. J. Am. Chem. Soc..

[156] Lee B., Lee D.G. (2019). Synergistic antibacterial activity of gold nanoparticles caused by apoptosis-like death. J. Appl. Microbiol..

[157] Mahdi H.S., Parveen A. (2019). Biosynthesis, Characterization and antibacterial activity of gold nanoparticles (Au-NPs) using black lemon extract. Mater. Today Proc..

[158] Dasari T.S., Zhang Y., Yu H. (2015). Antibacterial activity and cytotoxicity of gold (I) and (III) ions and gold nanoparticles. Biochem. Pharmacol. Open Access.

[159] Wang C., Cui Q., Wang X., Li L. (2016). Preparation of hybrid gold/polymer nanocomposites and their application in a controlled antibacterial assay. ACS Appl. Mater. Interfaces.

[160] Khan a.S., Bakht J., Syed F. (2018). Green synthesis of gold nanoparticles using Acer pentapomicum leaves extract its characterization, antibacterial, antifungal and antioxidant bioassay. Dig. J. Nanomater. Biostruct.

[161] Mahmoud N.N., Alhusban A.A., Ali J.I., Al-Bakri A.G., Hamed R., Khalil E.A. (2019). Preferential accumulation of phospholipid-PEG and cholesterol-PEG decorated gold nanorods into human skin layers and their photothermal-based antibacterial activity. Sci. Rep..

[162] Khedr W.E., Shaheen M.N., Elmahdy E.M., El-Bendary M.A., Hamed A.A., Mohamedin A.H. (2024). Silver and gold nanoparticles: Eco-friendly synthesis, antibiofilm, antiviral, and anticancer bioactivities. Prep. Biochem. Biotechnol..

[163] Aljarba N.H., Imtiaz S., Anwar N., Alanazi I.S., Alkahtani S. (2022). Anticancer and microbial activities of gold nanoparticles: A mechanistic review. J. King Saud. Univ.-Sci..

[164] Mi X.-j., Park H.-R., Dhandapani S., Lee S., Kim Y.-J. (2022). Biologically synthesis of gold nanoparticles using *Cirsium japonicum* var. *maackii* extract and the study of anti-cancer properties on AGS gastric cancer cells. Int. J. Biol. Sci..

[165] Muthukumar T., Sambandam B., Aravinthan A., Sastry T.P., Kim J.-H. (2016). Green synthesis of gold nanoparticles and their enhanced synergistic antitumor activity using HepG2 and MCF7 cells and its antibacterial effects. Process Biochem..

[166] Majumdar M., Biswas S.C., Choudhury R., Upadhyay P., Adhikary A., Roy D.N., Misra T.K. (2019). Synthesis of gold nanoparticles using citrus macroptera fruit extract: Anti-biofilm and anticancer activity. ChemistrySelect.

[167] Arunachalam K.D., Arun L.B., Annamalai S.K., Arunachalam A.M. (2014). Biofunctionalized gold nanoparticles synthesis from *Gymnema sylvestre* and its preliminary anticancer activity. Int. J. Pharm. Pharm. Sci..

[168] Wang L., Xu J., Yan Y., Liu H., Li F. (2019). Synthesis of gold nanoparticles from leaf *Panax notoginseng* and its anticancer activity in pancreatic cancer PANC-1 cell lines. Artif. Cells Nanomed. Biotechnol..

[169] Patil M.P., Jin X., Simeon N.C., Palma J., Kim D., Ngabire D., Kim N.-H., Tarte N.H., Kim G.-D. (2018). Anticancer activity of Sasa borealis leaf extract-mediated gold nanoparticles. Artif. Cells Nanomed. Biotechnol..

[170] Sun B., Hu N., Han L., Pi Y., Gao Y., Chen K. (2019). Anticancer activity of green synthesised gold nanoparticles from *Marsdenia tenacissima* inhibits A549 cell proliferation through the apoptotic pathway. Artif. Cells Nanomed. Biotechnol..

[171] Wu T., Duan X., Hu C., Wu C., Chen X., Huang J., Liu J., Cui S. (2019). Synthesis and characterization of gold nanoparticles from *Abies spectabilis* extract and its anticancer activity on bladder cancer T24 cells. Artif. Cells Nanomed. Biotechnol..

[172] Qian L., Su W., Wang Y., Dang M., Zhang W., Wang C. (2019). Synthesis and characterization of gold nanoparticles from aqueous leaf extract of *Alternanthera sessilis* and its anticancer activity on cervical cancer cells (HeLa). Artif. Cells Nanomed. Biotechnol..

[173] Govindaraju K., Vasantharaja R., Suganya K.U., Anbarasu S., Revathy K., Pugazhendhi A., Karthickeyan D., Singaravelu G. (2020). Unveiling the anticancer and antimycobacterial potentials of bioengineered gold nanoparticles. Process Biochem..

[174] Kajani A.A., Bordbar A.-K., Esfahani S.H.Z., Razmjou A. (2016). Gold nanoparticles as potent anticancer agent: Green synthesis, characterization, and in vitro study. RSC Adv..

[175] Tan G., Tevlek A., Aydin H.M. (2023). Comparison of garlic and onion extract-derived gold nanoparticles: Characterization and anticancer activity. J. Drug Deliv. Sci. Technol..

[176] Palaniyandi T., Viswanathan S., Prabhakaran P., Baskar G., Wahab M.R.A., Sivaji A., Ravi M., Rajendran B.K., Moovendhan M., Surendran H. (2023). Green synthesis of gold nanoparticles using *Halymenia pseudofloresii* extracts and their antioxidant, antimicrobial, and anti-cancer activities. Biomass Convers. Biorefinery.

[177] Abdulateef S., Raypah M.E., Omar A., Jafri M.M., Ahmed N.M., Kaus N.H.M., Seeni A., Mail M.H., Tabana Y., Ahmed M. (2023). Rapid synthesis of bovine serum albumin-conjugated gold nanoparticles using pulsed laser ablation and their anticancer activity on hela cells. Arab. J. Chem..

[178] Viswanathan S., Palaniyandi T., Chellam D.C., Ahmed M.F., Shoban N., Pushpakumar M., Wahab M.R.A., Baskar G., Ravi M., Sivaji A. (2023). Anti-cancer activity of *Hypnea valentiae* seaweed loaded gold nanoparticles through EMT signaling pathway in A549 cells. Biochem. Syst. Ecol..

[179] Gawali P., Ramteke L., Jadhav B., Khade B.S. (2023). Trypsin conjugated Au nanoparticles using *Sonneratia alba* fruits: Interaction and binding studies with antioxidant, anti-inflammatory, and anticancer activities. J. Clust. Sci..

[180] Nisa S.A., Govindaraju K., Vasantharaja R., Kannan M., Karthickeyan D. (2023). Biosynthesis of gold nanoparticles using jellyfish nematocyst venom protein and evaluation of their anticancer activity against breast cancer cells: In-vitro study. Chem. Phys. Lett..

[181] Pomal N.C., Bhatt K.D., Kundariya D.S., Desai R.A., Bhatt V., Kongor A. (2023). Calix [4] pyrrole-Grafted Gold Nanoparticles as a Turn-On Fluorescence Sensor for Noxious Fungicide Dimoxystrobin and Their Anti-Cancer Activity against the KB-3-1 Cell Line. ChemistrySelect.

[182] Susanna D., Balakrishnan R.M., Ettiyappan J.P. (2023). Ultrasonication-assisted green synthesis and characterization of gold nanoparticles from *Nothapodytes foetida*: An assessment of their antioxidant, antibacterial, anticancer and wound healing potential. J. Drug Deliv. Sci. Technol..

[183] Hammad S.E., El-Rouby M.N., Abdel-Aziz M.M., El-Sayyad G.S., Elshikh H.H. (2025). Endophytic fungi–assisted biomass synthesis of gold, and zinc oxide nanoparticles for increasing antibacterial, and anticancer activities. Biomass Convers. Biorefinery.

[184] Das C.A., Kumar V.G., Dharani G., Dhas T.S., Karthick V., Kumar C.V., Embrandiri A. (2023). Macroalgae-associated halotolerant marine bacteria *Exiguobacterium aestuarii* ADCG SIST3 synthesized gold nanoparticles and its anticancer activity in breast cancer cell line (MCF-7). J. Mol. Liq..

[185] Singh A.K., Tiwari R., Singh V.K., Singh P., Khadim S.R., Singh U., Srivastava V., Hasan S., Asthana R. (2019). Green synthesis of gold nanoparticles from *Dunaliella salina*, its characterization and in vitro anticancer activity on breast cancer cell line. J. Drug Deliv. Sci. Technol..

[186] Ajdari Z., Rahman H., Shameli K., Abdullah R., Abd Ghani M., Yeap S., Abbasiliasi S., Ajdari D., Ariff A. (2016). Novel gold nanoparticles reduced by *Sargassum glaucescens*: Preparation, characterization and anticancer activity. Molecules.

[187] Mmola M., Roes-Hill M.L., Durrell K., Bolton J.J., Sibuyi N., Meyer M.E., Beukes D.R., Antunes E. (2016). Enhanced antimicrobial and anticancer activity of silver and gold nanoparticles synthesised using *Sargassum incisifolium* aqueous extracts. Molecules.

[188] Ye H., Shen Z., Yu L., Wei M., Li Y. (2018). Manipulating nanoparticle transport within blood flow through external forces: An exemplar of mechanics in nanomedicine. Proc. R. Soc. A Math. Phys. Eng. Sci..

[189] Kim C.K., Ghosh P., Pagliuca C., Zhu Z.-J., Menichetti S., Rotello V.M. (2009). Entrapment of hydrophobic drugs in nanoparticle monolayers with efficient release into cancer cells. J. Am. Chem. Soc..

[190] Puja A.M., Xu X., Wang R., Kim H., Kim Y.-J. (2022). Ginsenoside compound K-loaded gold nanoparticles synthesized from *Curtobacterium proimmune* K3 exerts anti-gastric cancer effect via promoting PI3K/Akt-mediated apoptosis. Cancer Nanotechnol..

[191] Patil M.P., Kim G.-D. (2017). Eco-friendly approach for nanoparticles synthesis and mechanism behind antibacterial activity of silver and anticancer activity of gold nanoparticles. Appl. Microbiol. Biotechnol..

[192] Bajracharya R., Song J.G., Patil B.R., Lee S.H., Noh H.-M., Kim D.-H., Kim G.-L., Seo S.-H., Park J.-W.-W., Jeong S.H. (2022). Functional ligands for improving anticancer drug therapy: Current status and applications to drug delivery systems. Drug Deliv..

[193] Tan K.F., In L.L.A., Vijayaraj Kumar P. (2023). Surface functionalization of gold nanoparticles for targeting the tumor microenvironment to improve antitumor efficiency. ACS Appl. Bio Mater..

[194] Nisha, Sachan R.S.K., Singh A., Karnwal A., Shidiki A., Kumar G. (2024). Plant-mediated gold nanoparticles in cancer therapy: Exploring anti-cancer mechanisms, drug delivery applications, and future prospects. Front. Nanotechnol..

[195] Magogotya M., Vetten M., Roux-van der Merwe M., Badenhorst J., Gulumian M. (2022). In vitro toxicity and internalization of gold nanoparticles (AuNPs) in human epithelial colorectal adenocarcinoma (Caco-2) cells and the human skin keratinocyte (HaCaT) cells. Mutat. Res./Genet. Toxicol. Environ. Mutagen..

[196] Mitchell M.J., Billingsley M.M., Haley R.M., Wechsler M.E., Peppas N.A., Langer R. (2021). Engineering precision nanoparticles for drug delivery. Nat. Rev. Drug Discov..

[197] Gong N., Chen S., Jin S., Zhang J., Wang P.C., Liang X.-J. (2015). Effects of the physicochemical properties of gold nanostructures on cellular internalization. Regen. Biomater..

[198] Cui W., Li J., Zhang Y., Rong H., Lu W., Jiang L. (2012). Effects of aggregation and the surface properties of gold nanoparticles on cytotoxicity and cell growth. Nanomed. Nanotechnol. Biol. Med..

[199] Dhandapani S., Xu X., Wang R., Puja A.M., Kim H., Perumalsamy H., Balusamy S.R., Kim Y.-J. (2021). Biosynthesis of gold nanoparticles using *Nigella sativa* and *Curtobacterium proimmune* K3 and evaluation of their anticancer activity. Mater. Sci. Eng. C.

[200] Patil M.P., Ngabire D., Thi H.H.P., Kim M.-D., Kim G.-D. (2017). Eco-friendly synthesis of gold nanoparticles and evaluation of their cytotoxic activity on cancer cells. J. Clust. Sci..

[201] Jeyaraj M., Arun R., Sathishkumar G., MubarakAli D., Rajesh M., Sivanandhan G., Kapildev G., Manickavasagam M., Thajuddin N., Ganapathi A. (2014). An evidence on G2/M arrest, DNA damage and caspase mediated apoptotic effect of biosynthesized gold nanoparticles on human cervical carcinoma cells (HeLa). Mater. Res. Bull..

[202] Tiloke C., Phulukdaree A., Anand K., Gengan R.M., Chuturgoon A.A. (2016). Moringa oleifera gold nanoparticles modulate oncogenes, tumor suppressor genes, and caspase-9 splice variants in a549 cells. J. Cell. Biochem..

